# Variation in HIV-1 Tat activity is a key determinant in the establishment of latent infection

**DOI:** 10.1172/jci.insight.184711

**Published:** 2024-12-05

**Authors:** Francisco Gomez-Rivera, Valeri H. Terry, Cuie Chen, Mark M. Painter, Maria C. Virgilio, Marianne E. Yaple-Maresh, Kathleen L. Collins

**Affiliations:** 1 Graduate Program in Immunology,; 2 Department of Internal Medicine,; 3 Department of Computational Medicine and Bioinformatics,; 4 Cellular and Molecular Biology Program, and; 5 Department of Microbiology & Immunology, University of Michigan Medical School, Ann Arbor, Michigan, USA.

**Keywords:** Infectious disease, Virology, Cellular immune response, T cells

## Abstract

Despite effective treatment, human immunodeficiency virus (HIV) persists in optimally treated people as a transcriptionally silent provirus. Latently infected cells evade the immune system and the harmful effects of the virus, thereby creating a long-lasting reservoir of HIV. To gain a deeper insight into the molecular mechanisms of HIV latency establishment, we constructed a series of HIV-1 fluorescent reporter viruses that distinguish active versus latent infection. We unexpectedly observed that the proportion of active to latent infection depended on a limiting viral factor, which created a bottleneck that could be overcome by superinfection of the cell, T cell activation, or overexpression of HIV-1 transactivator of transcription (Tat). In addition, we found that *tat* and regulator of expression of virion proteins (Rev) expression levels varied among HIV molecular clones and that *tat* levels were an important variable in latency establishment. Lower *rev* levels limited viral protein expression whereas lower Tat levels or mutation of the Tat binding element promoted latent infection that was resistant to reactivation even in fully activated primary T cells. Nevertheless, we found that combinations of latency reversal agents targeting both cellular activation and histone acetylation pathways overcame deficiencies in the Tat/TAR axis of transcription regulation. These results provide additional insight into the mechanisms of latency establishment and inform Tat-centered approaches to cure HIV.

## Abstract 2

Our studies utilized unique dual-reporter probes that revealed a natural deficiency in Tat expression that primes HIV for the establishment of silent infections that can evade the immune response and lead to persistent infections.

## Graphical abstract

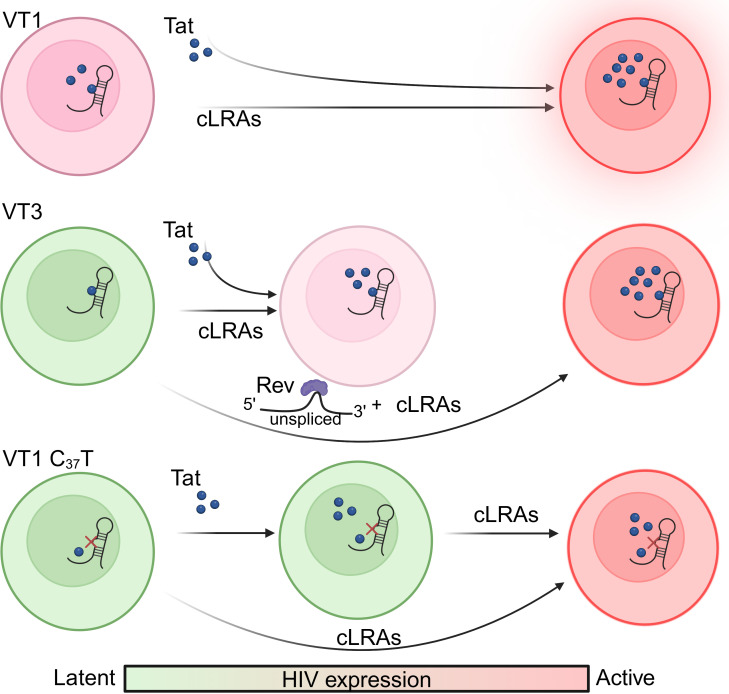

## Introduction

Human immunodeficiency virus (HIV) is a lentivirus characterized by its ability to establish chronic infection by evading the immune system. Since the beginning of the pandemic, HIV has infected more than 85.6 million people worldwide (1). Currently, more than 39 million people are living with HIV, only 29.8 million of whom have access to antiretroviral therapy (1). Combination antiretroviral therapy (cART) inhibits HIV spread and reduces transmission as well as the onset of immunodeficiency. However, it is costly for patients, with direct and indirect costs totaling $1 million per infected individual (2). In addition, antiretroviral therapy is not a cure, as it does not eradicate latently infected cells that are long-lived and people must remain on therapy for life (3, 4). While actively infected cells have a short half-life (5) and eventually decline, latently infected cells persist for decades (6). Thus, latently infected cells form the majority of the remaining viral reservoir in people who have been optimally treated for years (5, 7–9). Consequently, there is an urgent need to understand HIV latency and identify strategies that can eradicate latent reservoirs.

Because actively infected cells have a short half-life because of the toxic effects of the virus and the anti-HIV immune response, reactivation of latent reservoirs may provide a pathway to a cure. Thus, there is an urgent need to better understand viral and cellular factors that determine whether a viral infection will achieve a latent state. Studies thus far have shown that HIV latency results in part from recruitment of histone deacetylases (HDACs) that induce epigenetic modifications and heterochromatin structure that limits access of transcription factors to the viral long terminal repeat (LTR) (10). Treatment of latently infected cells with histone deacetylase inhibitors (HDACi) leads to decondensation of chromatin, increased accessibility of the HIV LTR, and viral reactivation (11). However, the effectiveness of HDACi at decreasing latent reservoirs in people is not sufficient to yield a clinical benefit, even in combination with anti-HIV T cells expanded ex vivo (12). Thus, additional strategies are needed to reverse latency in vivo and provide improved therapies for people living with HIV.

Latency establishment is also influenced by viral genetic elements and gene products. HIV transcriptional activity is driven by the LTR, which includes binding sites for cellular transcription factors, such as nuclear factor-κB (NF-κB) and activator protein 1 (reviewed in ref. 13). In quiescent T cells, NF-κB is sequestered in the cytoplasm (14), and in cell line models of latency, HIV gene transcription can be induced through NF-κB activation (15). However, in resting memory primary T cells, activation of positive transcription elongation factor b (P-TEFb) is also required (10).

HIV transactivator of transcription (Tat) is a pleiotropic HIV protein that is necessary for HIV gene expression, but the extent to which it plays a role in the establishment of HIV latency is not well understood. Tat binds to the HIV TAR element and recruits P-TEFb (16–18), which increases RNA polymerase processivity (19) by phosphorylation of RNA polymerase cytoplasmic tail domain (19). Tat also interacts with nuclear histone acetyltransferases (HATs) (20–22) and the SWI/SNF chromatin-remodeling complex (23, 24), which increases viral transcription by altering chromatin structure around the LTR (25). There is also evidence that Tat interacts with transcription factors and/or signal transduction pathways that promote LTR transcriptional activity (26–30).

To allow the virus to pack multiple functions into a small genetic space, HIV encodes a second regulatory protein (Rev) from a reading frame that overlaps with Tat. Rev facilitates the export of unspliced and partially spliced viral RNAs from the nucleus to the cytoplasm. Rev binds to a highly structured RNA sequence known as the Rev response element (RRE) located within the envelope (*env*) region of the viral genome (31). By interacting with the host’s nuclear export machinery, Rev ensures that these viral transcripts are appropriately translated into essential structural proteins and enzymes required for assembling new virus particles (32).

To better study the process of latency establishment, a number of labs have generated fluorescent reporter viruses that distinguish active versus latent infection (33–39). Here, we report a series of HIV-1 latency reporter constructs derived from proviral genomes isolated from people infected with HIV at different disease stages. Two constructs (89.6 VT1 and 89.6 VT3) were derived from a person with AIDS (40). The third construct (454 VT2) was derived from a person on cART with undetectable levels of plasma virus (41). All 3 reporters allow the identification and isolation of latently infected cells but differ in the extent to which they support latency.

Using these probes, we consistently observed that the likelihood of latency establishment decreased as virus inoculum increased. Mechanistic studies revealed that a limitation in HIV-1 Tat expression at low virus inoculum was the most likely explanation for the high proportion of latent infection. In addition, the probes we constructed differed in their relative ability to express *tat* and *rev*. Variation in Tat modulated the extent to which latency was established, whereas variation in both Tat and Rev affected responsiveness to latency reversal agents (LRAs) as assessed by reporter protein expression. Unexpectedly, however, we found that even profound Tat deficiency could be overcome by increasing the number and type of LRAs that inhibit histone deacetylation and stimulate transcription factor activation. Thus, the role of Tat appears to be mechanistically limited to the pathways these agonists activate. These results provide additional insight into mechanisms of latency establishment and inform Tat-centered approaches to cure HIV.

## Results

### Dual reporter 89.6 VT1 can distinguish latent and active HIV gene expression in CEM-SS cells.

**Figure 1:**
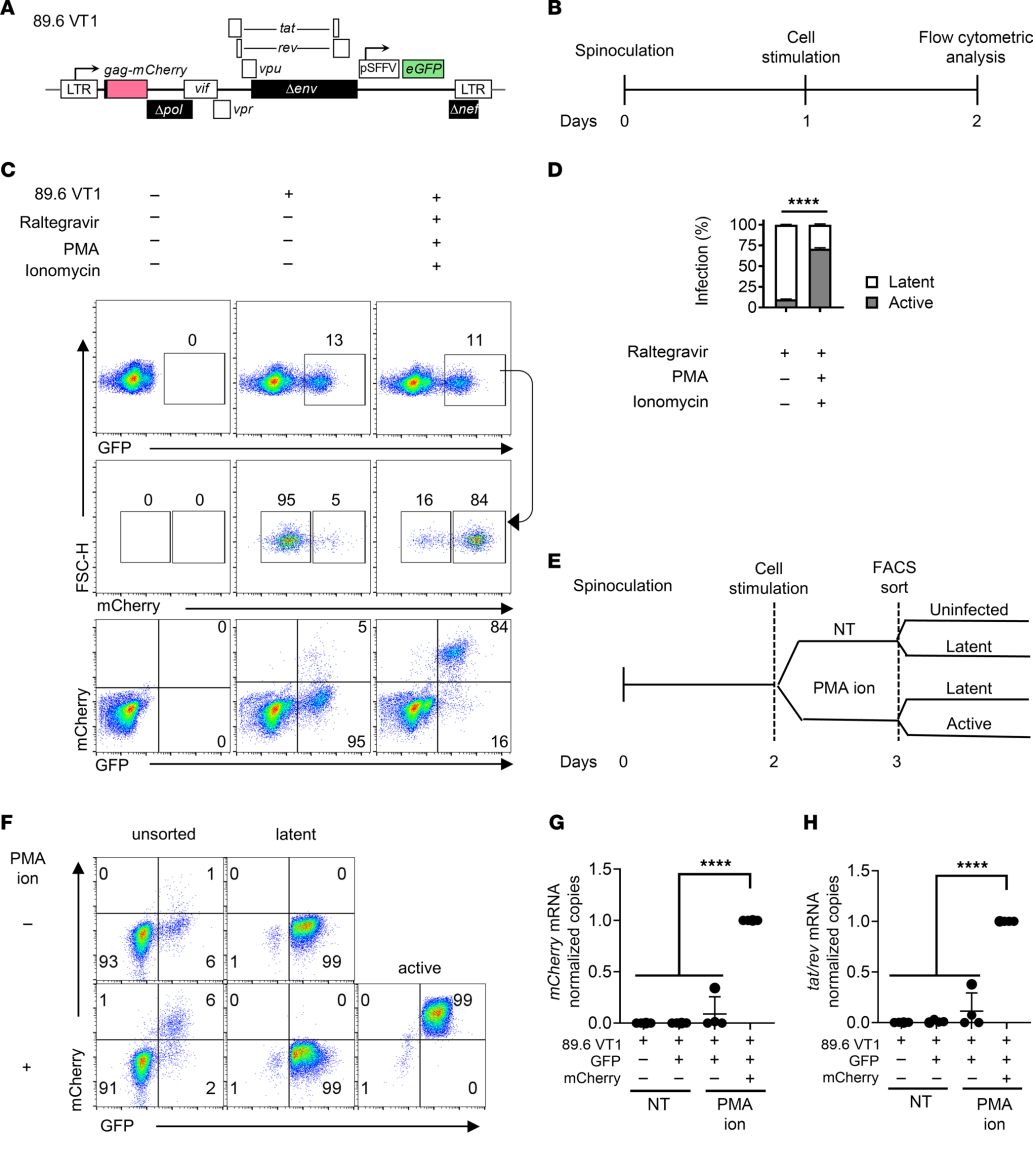
Dual reporter 89.6 VT1 can distinguish between latent and active HIV gene expression in CEM-SS cells. (**A**) Diagram for dual reporter 89.6 VT1 expressing mCherry as a Gag-mCherry fusion protein using the native HIV promoter and *eGFP* driven by the spleen focus forming virus promoter (pSFFV) inserted in the *env* and *nef* open reading frames. (**B**) Schematic demonstrating the experimental process for **C** and **D**. (**C**) Flow cytometric analysis of CEM-SS cells transduced with 89.6 VT1 and treated with PMA, ionomycin, and raltegravir as indicated according to the timeline shown in **B**. (**D**) Summary graph of flow cytometric data for CEM-SS cells treated as for **C**. Statistical significance was determined by 2-way ANOVA with Holm-Šídák multiple comparisons test. The mean ± standard deviation is shown for 4 independent experiments, ****P ≤ 0.0001. (**E**) Schematic of the experimental process for panels **F**–**H**. (**F**) Flow cytometric analyses of CEM-SS cells transduced with 89.6 VT1, treated as indicated, and sorted according to the timeline shown in **E**. (**G** and **H**) Summary graphs of RT-qPCR analysis of RNA from CEM-SS cells treated according to the timeline shown in **E** and isolated by FACS as in **F**. RNA copies were normalized to *GAPDH* RNA copies. Statistical significance was determined by 2-way ANOVA with Tukey’s multiple comparisons test (**G** and **H**). The mean ± standard deviation is shown for **G** and **H** for 4 independent experiments, ****P ≤ 0.0001. FSC, forward scatter; NT, no treatment.

To enhance our ability to understand factors that influence the establishment of HIV latency, we developed a dual reporter HIV (89.6 VT1; Figure 1A). The parental virus of the reporter (HIV 89.6) was isolated from the peripheral blood of an HIV-infected person with AIDS (40). The 89.6 VT1 “latency probe” expresses GFP from the “constitutive” spleen focus forming virus (SFFV) promoter and mCherry from the HIV LTR. Hence, latently infected cells are GFP^+^mCherry^–^. Because the design of this reporter placed the constitutively active promoter within the negative effective factor (*nef*) open reading frame, this construct cannot express *nef*. Using this latency probe, we used flow cytometry to assess the extent to which CEM-SS, a human T cell lymphoma cell line, supported latent infection (Figure 1B). Nearly all the unstimulated CEM-SS cells were latently infected based on GFP and mCherry expression (95%; Figure 1, C and D). To verify that the probe identified latently infected cells that could be reactivated, we compared results from untreated cells to matched samples treated with LRAs that stimulate T cell activation pathways (phorbol myristate acetate [PMA] and ionomycin). To ensure changes in gene expression were not the result of new integration events, we included an integrase inhibitor (raltegravir) with the activation cocktail. Nearly all the cells that were latently infected (GFP^+^mCherry^–^), became actively infected (GFP^+^mCherry^+^) following treatment (Figure 1, C and D). In addition, we found that latency in this system could be reversed to varying degrees with other well-established LRAs (tumor necrosis factor-α [TNF-α], HDACis [vorinostat and entinostat], protein kinase C [PKC] agonists [bryostatin-1, ref. 42]; and the P-TEFb activator hexamethylene bisacetamide) (Supplemental Figure 1, A and B; supplemental material available online with this article; https://doi.org/10.1172/jci.insight.184711DS1). Thus, 89.6 VT1 appeared to function as a bona fide probe of HIV latency capable of quantifying the extent to which latency was reversed by different LRAs.

To determine whether latency as measured by the reporter virus was at the level of RNA transcription, CEM-SS cells treated with virus plus or minus PMA and ionomycin activation were isolated by fluorescence-activated cell sorting (FACS) into latent or active populations (Figure 1, E and F). Then, reverse transcription quantitative PCR (RT-qPCR) of RNA isolated from each population was performed using a primer/probe combination that selectively amplified spliced *tat* and *rev* (*tat/rev*) or *mCherry* transcripts. RT-qPCR analysis of *mCherry* (Figure 1G) and spliced *tat/rev* (Figure 1H) revealed that actively infected cells identified by *mCherry* expression had 11-fold higher *mCherry* RNA and 8-fold higher spliced *tat/rev* RNA relative to GFP^+^ cells that lacked mCherry protein. Thus, CEM-SS cells transduced with 89.6 VT1 dual reporter supported transcriptional latency that was reversible with PMA and ionomycin.

To examine whether CEM-SS cells transduced with 89.6 VT1 resulted in latency that was stable over time, CEM-SS cells were transduced with 89.6 VT1, sorted to isolate the latent population, and cultured for up to 21 days. As shown in Supplemental Figure 1, C and D, we observed that approximately three-quarters of the population maintained latent infection over this time period. An analysis of the actively infected population revealed that these cells diminished over time because of spontaneous reversion to latency (Supplemental Figure 2, A and B) and slower growth kinetics (Supplemental Figure 2C). We also noted that both promoters could be silenced with extended culture, leading to the accumulation of “double-negative” cells that harbored viral genomes (Supplemental Figure 3, A–C). In contrast double-negative cells that were isolated soon after infection were negative for HIV sequences (Supplemental Figure 3C). Double-negative cells harboring viral genomes were reactivated with PMA and ionomycin (Supplemental Figure 3D).

### 89.6 VT1 identifies reversible latency in primary human HSPCs.

**Figure 2:**
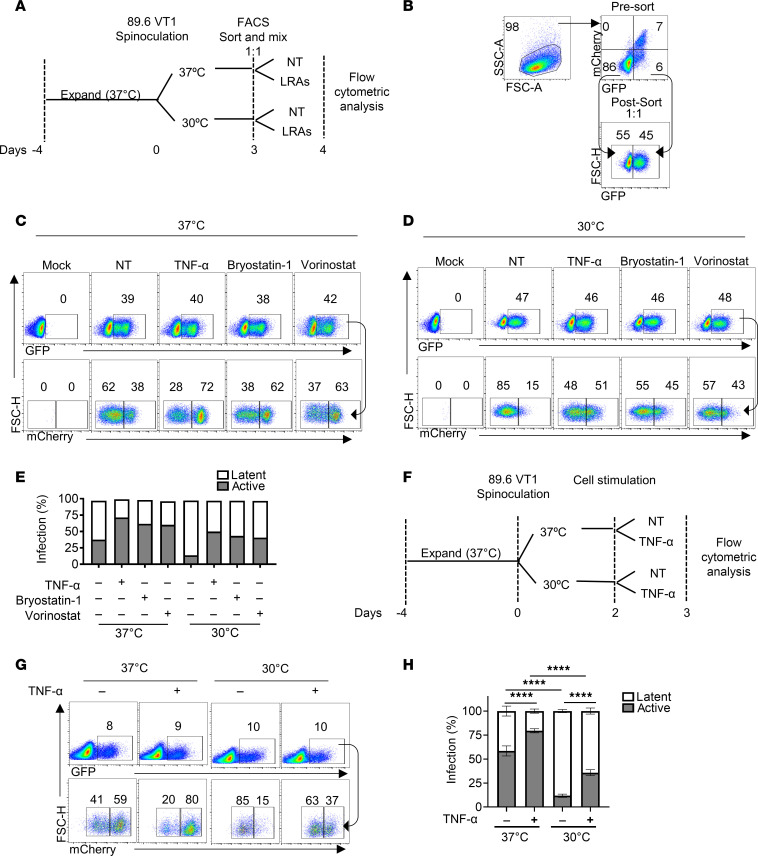
89.6 VT1 identifies reversible latency in primary human HSPCs. (**A**) Schematic of the experimental process for **B**–**E**. (**B**) Flow cytometric analysis of HSPCs expanded, transduced, and sorted according to the timeline shown in **A**. As indicated, actively infected cells were removed via FACS by sorting latently infected (GFP^+^mCherry^–^) cells. The isolated cells were mixed with uninfected cells so that changes in the proportions of active and latent infection following LRA treatment could be more accurately quantified. (**C** and **D**) Flow cytometric analysis of HSPCs from **B** divided into 37°C or 30°C incubation conditions with LRAs as indicated for 24 hours. (**E**) Summary graph of flow cytometric analysis (**C** and **D**). Result is shown for 1 experiment. (**F**) Schematic of the experimental process for **G** and **H**. (**G**) Flow cytometric analysis of HSPCs expanded and transduced according to the timeline shown in **F**. (**H**) Summary graph of flow cytometric analysis performed as in **G**. Statistical significance was determined by 2-way ANOVA with Holm-Šídák multiple comparisons test. The mean ± standard deviation is shown for 3 independent experiments. ****P ≤ 0.0001. SSC, side scatter.

In addition to CD4^+^ T cells, CD4^+^ hematopoietic stem and progenitor cells (HSPCs) are a potential viral reservoir (43). There is evidence that they can be infected in vivo and that they amplify integrated viral genomes by cellular proliferation and differentiation into a variety of cell types (41, 43, 44). In contrast with activated primary T cells, HSPCs can establish latency immediately upon infection and do not require reversion to a quiescent state (41, 45). While HSPCs naturally favor HIV latency, active infection can spontaneously occur upon differentiation that inevitably occurs even with optimal HSPC culture conditions. We have found that spontaneous activation of latent infection can be decreased by culturing HSPCs under hypothermic (30°C) conditions, which maintains a quiescent state that promotes latency (45). To determine whether 89.6 VT1 reliably detects latently infected HSPCs, we transduced HSPCs with 89.6 VT1 and isolated the latent (GFP^+^mCherry^–^) population by FACS. The sorted HSPCs were remixed with uninfected GFP^–^ HSPCs at a one-to-one ratio before being divided for incubation either at 30°C or at 37°C plus or minus LRAs (Figure 2, A and B). Consistent with our prior results (45), we observed more spontaneous activation of the latently infected HSPCs when they were incubated at 37°C as compared with 30°C (38% versus 15% in Figure 2, C and D, respectively). In addition, we observed the expected increase in active infection with each LRA tested (Figure 2, C and D, and summarized in Figure 2E). Similar results were also achieved without removing actively infected cells before stimulation (Figure 2, F–H). However, this protocol resulted in a higher level of active infection in the 37°C untreated condition that reduced the apparent effect of TNF-α stimulation. In sum, these data show that 89.6 VT1 dual reporter is suitable for studying HIV latency in HSPCs.

### Factors that determine the likelihood of active and latent infection in reporter viruses from different HIV molecular clones.

**Figure 3:**
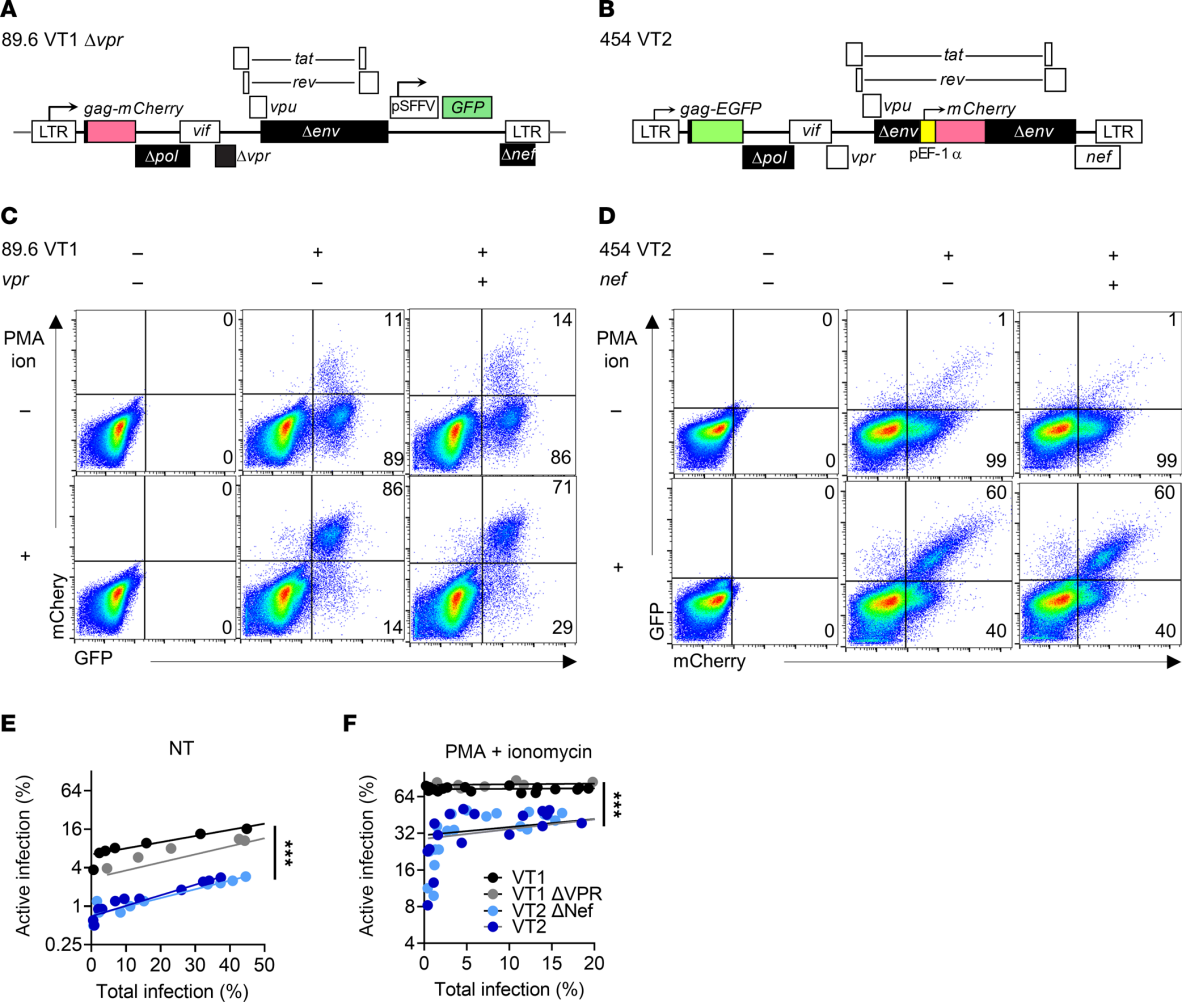
Factors that determine the likelihood of active and latent infection in reporter viruses from different HIV molecular clones. (**A**) Diagram for dual reporter 89.6 VT1 probe as described in Figure 1A legend. (**B**) Diagram for dual reporter 454 VT2 reporter expressing GFP as a Gag-eGFP fusion protein using the native HIV promoter and mCherry driven by the elongation factor 1-a (EF1-α) promoter inserted in *env*. (**C** and **D**) Flow cytometric analysis of CEM-SS cells transduced with the indicated reporter virus, treated with PMA and ionomycin (ion) as indicated at 2 days postinfection (dpi), and harvested 3 dpi. (The same mock sample was used for **C** and **D**, but the *x* and *y* axes were transposed to allow representation of active infection on the *y* axis.) (**E** and **F**) Summary graphs of flow cytometric data from CEM-SS cells transduced with increasing amounts of the indicated virus and treated where indicated with PMA and ionomycin as described for **C** and **D**. Each point represents a replicate from 1 experiment. Similar results were obtained in 3 independent experiments. Statistical significance was determined by Deming (Model II) linear regression (**E** and **F**). ***P ≤ 0.001.

Having determined that 89.6 VT1 can distinguish actively and latently infected T cells and HSPCs, we asked whether viral factors, such as viral protein R (Vpr) and Nef, which have been implicated in HIV transcription and T cell activation pathways (46–48), were playing a role in the establishment of HIV latency. To examine this, we modified 89.6 VT1 to create a version that was *vpr* null (Figure 3A). Western blot analysis revealed that Vpr expression was unexpectedly reduced in the 89.6 VT1 reporter as compared with its parent (Supplemental Figure 4A). Therefore, we also performed studies with wild-type 89.6 and its corresponding Vpr mutant (49) (Supplemental Figure 4, B–D).

89.6 VT1 has a constitutively active promoter inserted within the *nef* open reading frame and cannot express Nef. Thus, we developed a second HIV dual reporter that allowed all HIV accessory proteins to be expressed (454 VT2; Figure 3B). 454 VT2 is derived from a molecular clone that was PCR-amplified from a person with HIV on optimal cART (41). To preserve Nef expression, the constitutive reporter was placed within the *env* open reading frame after removing 709 bp of *env* (Figure 3B). Additional changes relative to 89.6 VT1 are that 454 VT2 has an mCherry reporter expressed from the “constitutive” (EF1-α) promoter; hence, latently infected cells are mCherry^+^eGFP^–^. Nef-negative versions of this construct were created by digesting and filling in a unique XhoI site within the *nef* open reading frame, which disrupted Nef activity as assessed by Nef-dependent MHC-I downmodulation in primary T cells (Supplemental Figure 4, E–H).

Initial studies comparing the pattern of latency establishment for each version of 454 VT2 relative to 89.6 VT1 verified that all reporters measured latent infection that could be reversed with PMA and ionomycin (Figure 3, C and D). However, we unexpectedly observed that the proportion of active to latent infection increased with increasing virus inoculum (Figure 3E). Although this was observed in untreated transduced CEM-SS cells, we found that stimulation with PMA and ionomycin greatly reduced the effect of viral inoculum, shifting the balance toward active infection regardless of the total infection rate (Figure 3F). We also noted that under all conditions tested, 454 VT2 favored latent infection relative to 89.6 VT1 (Figure 3, E and F).

Because virus inoculum influenced the proportion of active versus latent infection, comparison of mutant versus wild-type reporters was examined considering total infection rates (Figure 3, E and F). When active infection rates were compared at similar proportions of total infection, it was clear that *nef*-negative and *vpr-*negative dual reporters had a similar likelihood of establishing latent versus active infection as their respective parental dual reporters (Figure 3, E and F) in CEM-SS cells. Additionally, studies using the parental 89.6 virus verified that wild-type levels of Vpr did not affect latency in the CEM-SS latency model system (Supplemental Figure 4, C and D).

In sum, these studies verified that: (i) CEM-SS cells supported latent infection by wild-type HIV, (ii) the effect of viral inoculum on the likelihood of establishing active versus latent infection extended to wild-type virus, and (iii) 89.6 Vpr expression does not effect the establishment of latent versus active infection (Supplemental Figure 4, C and D). The marked impact of viral inoculum on active infection of wild-type and reporter viruses in unstimulated CEM-SS cells supports the hypothesis that another viral factor influenced the likelihood that latent infection was established.

### Variation in 5′ LTR sequences helps determine the proportion of actively versus latently infected quiescent CEM-SS cells.

**Figure 4:**
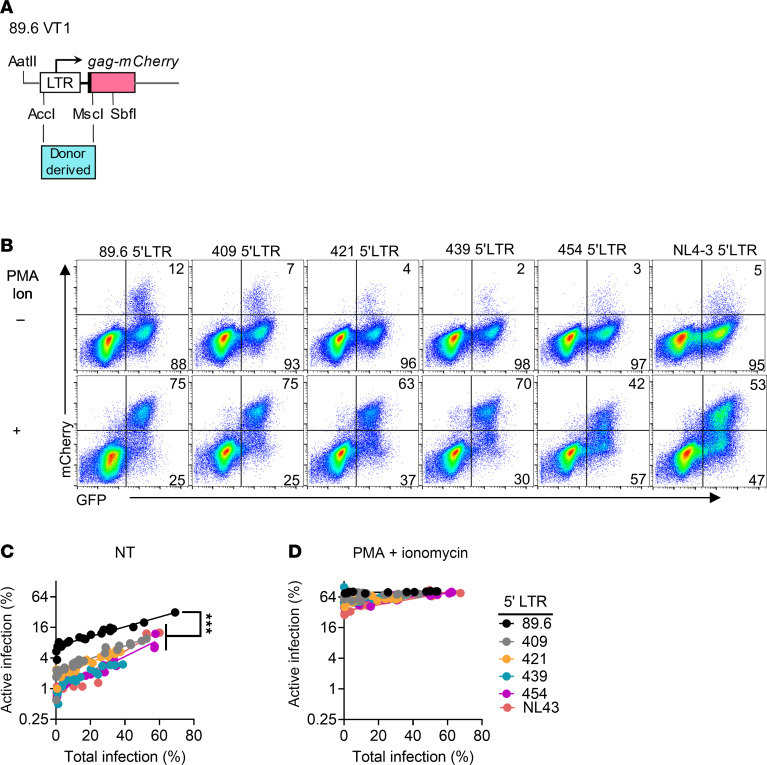
Variation in 5′ LTR sequence determines the proportion of actively versus latently infected quiescent CEM-SS cells. (**A**) Diagram displaying the location in which donor-derived 5′ LTR sequences corresponding to HXB2 position 39 to 806 were substituted for the corresponding region in the 89.6 5′ LTR of VT1. (**B**) Flow cytometric analysis of CEM-SS cells transduced with 89.6 VT1 containing the indicated donor-derived 5′ LTR sequences, treated with PMA and ionomycin as indicated 2 dpi, and harvested 3 dpi. (**C** and **D**) Summary graphs of experiments performed as in **B** with increasing amounts of each reporter virus. Each point represents a technical replicate from 1 independent experiment. Similar results were obtained in 6 independent experiments. (Two of these experiments included VT1 and VT1Δ*vpr* referred to in Figure 3.) Statistical significance was determined by Deming (Model II) linear regression (**C** and **D**). ***P ≤ 0.001.

Because transcription factor binding sites reside in the 5′ LTR, we next investigated whether genetic variations in the HIV-1 5′ regulatory region contribute to viral latency. To examine this, we identified LTR sequences from nearly full-length HIV proviral genomes from HSPCs isolated from 4 donors who were receiving cART and had clinically undetectable viral loads (41). Sequence variations occurred throughout the LTR region obtained from these genomes (Supplemental Figure 5). We replaced the 89.6 VT1 5′ LTR sequence with donor-derived sequences and used the resulting chimeric dual reporters to assess whether LTR sequence variations influenced the likelihood of latency establishment in CEM-SS cells (Figure 4A). We observed an increase in the likelihood of latency establishment with all 4 cART-treated patient–derived 5′ LTR VT1 constructs compared with 89.6 VT1 (Figure 4, B and C). As shown in Figure 4D, PMA and ionomycin reversed the latency of all cART-treated patient–derived 5′ LTR VT1 to the same degree as wild-type 89.6 VT1. Thus, the data indicate that sequence variation in the 5′ LTR region may be a factor in the establishment of latent versus active infection. Moreover, a relatively small (2.5-fold), statistically significant difference between 89.6 and 454 LTR may contribute to the substantial differences in latent versus active infection observed for the reporter constructs 89.6 VT1 and 454 VT2 shown in Figure 3E.

### HIV tat/rev levels vary in HIV molecular clones and reporter constructs.

**Figure 5:**
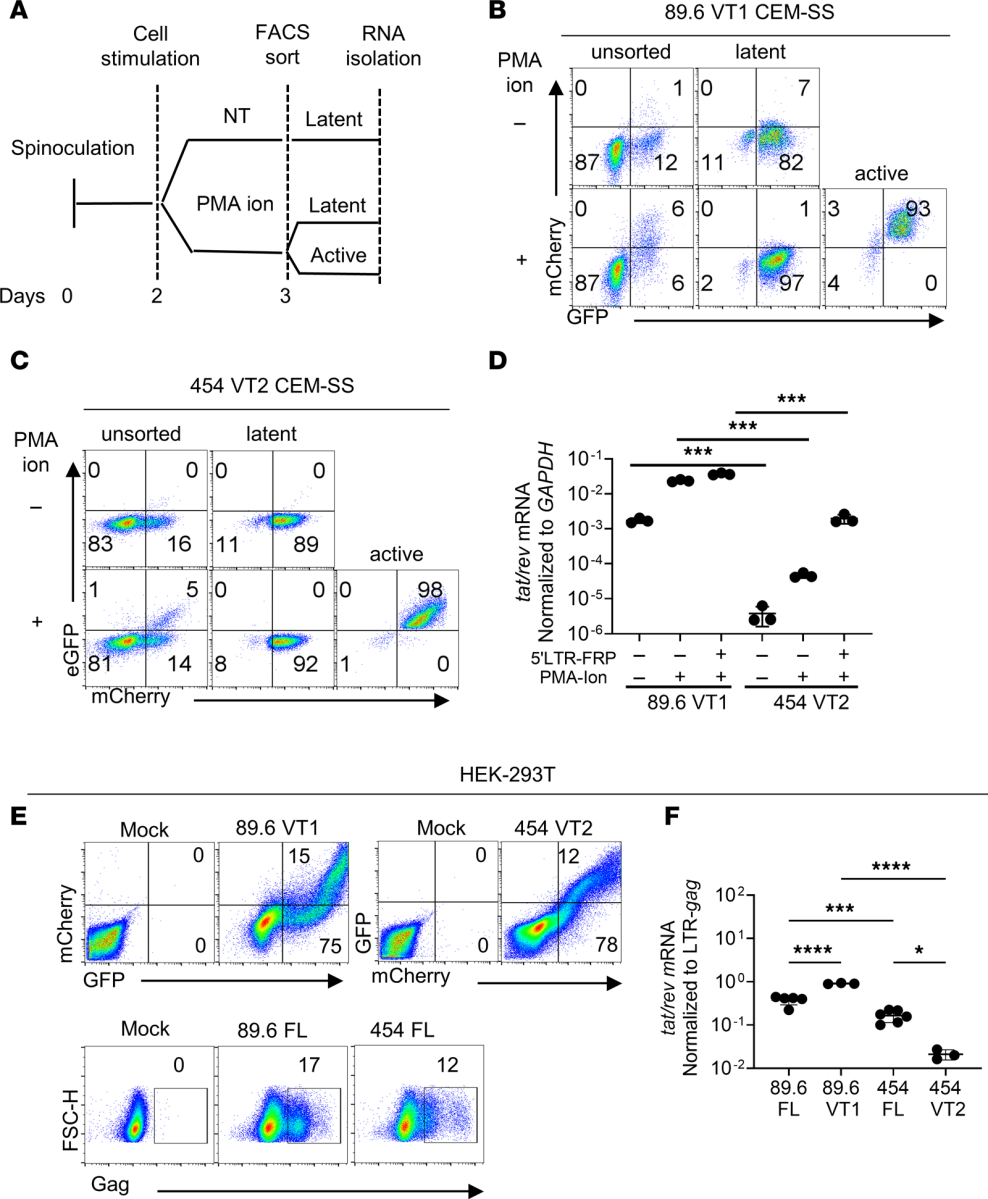
HIV *tat* levels vary in HIV molecular clones and reporter constructs. (**A**) Schematic of the experimental process for panels **B**–**D**. (**B** and **C**) Flow cytometric analysis of CEM-SS transduced with the indicated reporter virus, treated as indicated with PMA and ionomycin, and sorted according to the timeline shown in **A**. (**D**) Summary graph of RT-qPCR analysis of RNA isolated from CEM-SS cells treated with the indicated virus and sorted for latent or active infection according to the timeline shown in **A**. 5′ LTR-FRP indicates whether the fluorescent reporter protein (FRP) was expressed by each sorted population. Statistical significance was determined by 1-way ANOVA with Tukey’s multiple comparisons test. Mean values ± standard deviation are shown from 3 independent experiments, ***P ≤ 0.001. (**E**) Flow cytometric analysis of HEK293T cells transiently transfected with the indicated construct. Where indicated, permeabilized cells were stained with an antibody directed at HIV Gag. (The same mock sample was used for both viruses, but the *x* and *y* axes were transposed to allow representation of active infection on the *y* axis.) (**F**) Summary graph of RT-qPCR analysis of RNA from HEK293T cells transiently transfected with the indicated construct as in **E**. Statistical significance was determined by 1-way ANOVA with Tukey’s multiple comparisons test. Mean values ± standard deviation are shown from 3–5 independent experiments, *P ≤ 0.05; ***P ≤ 0.001; and ****P ≤ 0.0001.

Because mutations in Tat and its binding element (TAR) promote latency (50), we asked whether Tat levels varied between the 2 reporter viruses. In addition to their derivation from different HIV molecular clones, the location of the 454 VT2 constitutive promoter differs from 89.6 VT1 in that it lies between the 2 *tat* exons and thus might affect *tat* splicing and gene expression. To initially assess potential differences in *tat* gene expression, RT-qPCR was performed with a primer/probe combination that selectively amplified spliced *tat/rev* but that was unable to distinguish *tat* from *rev* because of their overlapping open reading frames. For these studies, we transduced cells at a low multiplicity of infection such that most of the untreated cells were latently infected (Figure 5, A–C). For comparison, we also sorted cell populations based on fluorescent reporter protein (FRP) expression following PMA and ionomycin treatment. RT-qPCR analysis revealed a striking 457-fold difference in *tat/rev* levels between untreated cells expressing 89.6 VT1 and 454 VT2 (Figure 5D). Treatment with PMA and ionomycin increased *tat/rev* mRNA expression dramatically for both reporters, even in cells that had not yet begun expressing the FRP (Figure 5D). However, this treatment did not fully reverse differences in *tat/rev* expression between 89.6 VT1 and 454 VT2 (Figure 5D).

Given our findings that *tat/rev* expression is an important variable among our constructs that plays an important role in determining latency establishment, we asked whether 89.6 and 454 parental HIV molecular clones similarly differ in *tat/rev* expression. Because these full-length, unmodified viral genomes lack fluorescent markers and have distinct entry requirements for infection, these assessments were performed in HEK293T cells transfected with the indicated plasmid DNA (Figure 5, E and F). As shown in Figure 5F, *tat/rev* expression level varied significantly among these constructs, even after adjusting for transfection by normalizing to *gag* (or *gag-mCherry*) expression. 89.6 VT1 demonstrated the highest relative expression followed by 89.6 wild-type, 454 wild-type, and 454 VT2. Interestingly, the changes in *tat/rev* expression for the reporter viral constructs relative to the respective wild-type parental HIV were in opposite directions, with 89.6 VT1 *tat/rev* expression increasing and 454 VT2 *tat/rev* decreasing. This can potentially be explained by changes in the intron size between the 2 *tat/rev* exons. A deletion in 89.6 VT1 decreased intron size by 702 bp whereas substitution with a constitutive promoter for the deleted sequence increased intron size by 225 bp for 454 VT2. Nevertheless, significant differences (2.3-fold) in *tat/rev* expression between 89.6 and 454 wild-type HIV molecular clones (Figure 5F) indicate that natural HIVs can vary significantly in *tat/rev* expression, potentially explained in part by differences in LTR activity observed in Figure 4. In addition, we verified that variations in both *tat* and *rev* occurred in parental viruses using primer probe sets that distinguished *tat* from *rev* transcripts (Supplemental Figure 6). Confirmatory immunoblot analysis was confounded by amino acid sequence variations between 89.6 and 454 Tat. Nevertheless, the results were consistent with the PCR data in that VT2 Tat was expressed less well than VT1 Tat despite the antibody better matching the VT2 Tat epitope (Supplemental Figure 7).

### Insertion of a constitutive promoter between tat exons in 89.6 VT1 reduces Tat expression and increases latency.

**Figure 6:**
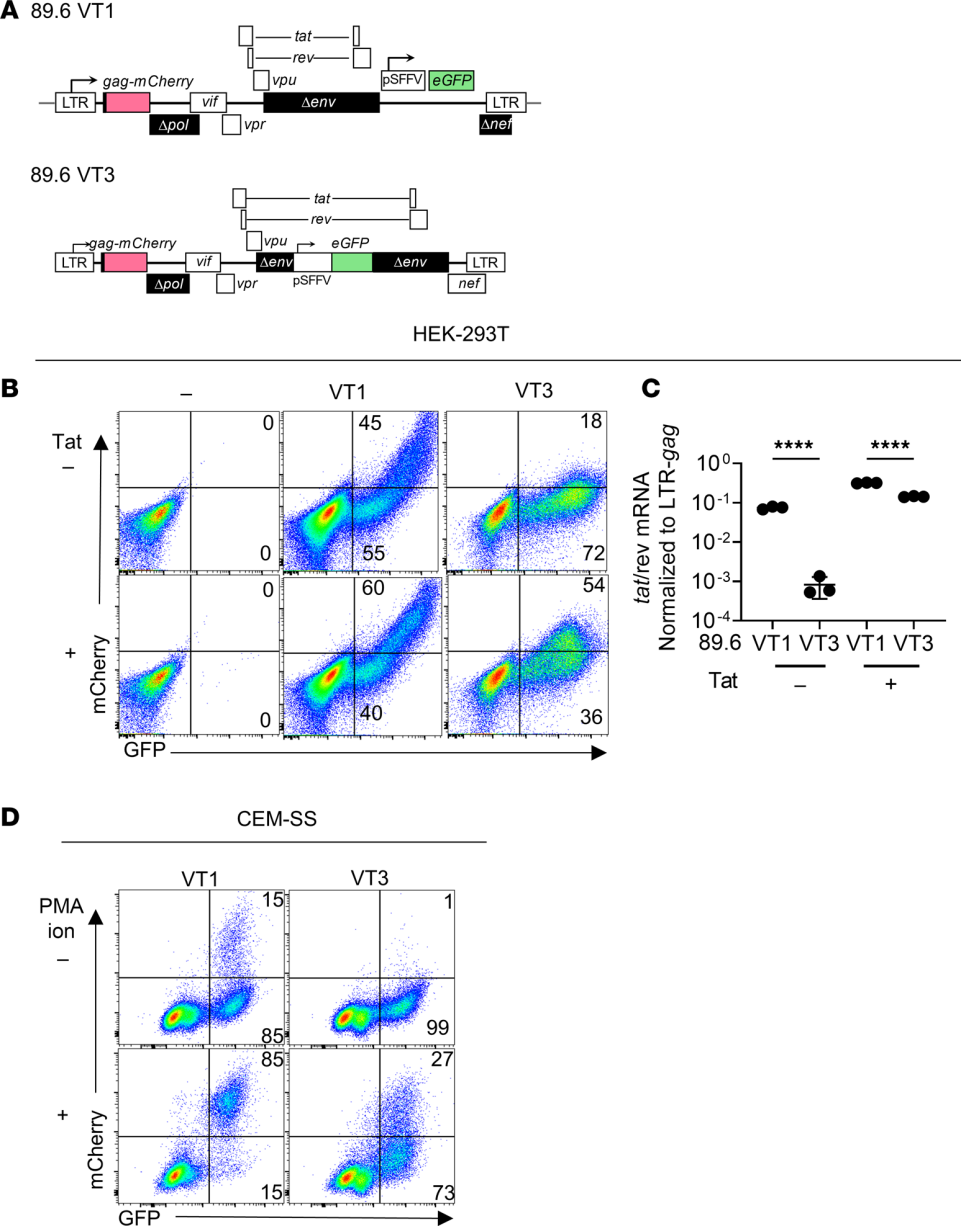
Insertion of constitutive promoter between Tat exons in 89.6 VT1 reduces Tat expression and increases latency. (**A**) Top, diagram of 89.6 VT1 as described in Figure 1A legend. Bottom, diagram for dual reporter, 89.6 VT3, in which *eGFP* driven by the spleen focus forming virus promoter (pSFFV) was inserted in *env* between *tat/rev* exons instead of 3′ to the second *tat* exon as in 89.6 VT1. (**B**) Flow cytometric analysis of HEK293T cells transiently transfected with the indicated reporter construct plus a plasmid expressing *tat*^89.6^ as indicated. (**C**) Summary graph of *tat/rev* RT-qPCR analysis of RNA isolated from HEK293T cells transiently transfected as for panel **B**. Statistical significance was determined by 1-way ANOVA with Tukey’s multiple comparisons test comparisons. Mean values ± standard deviation are shown, from 3 independent experiments, ****P ≤ 0.0001. (**D**) Flow cytometric analysis of CEM-SS cells transduced with the indicated reporter virus, treated 2 dpi as indicated with PMA and ionomycin, and harvested 3 dpi. Similar results were obtained in 3 independent experiments.

Given that insertions between the *tat/rev* exons in the HIV genome correlated with low *tat/rev* expression and increased latency in 454 VT2 compared with 89.6 VT1, we sought to extend this correlation using the same HIV molecular clone backbone (89.6). We hypothesized that insertion of a promoter element between the *tat/rev* exons of 89.6 VT1 (analogous to 454 VT2) would reduce *tat/rev* compared with unmodified 89.6 VT1. This also allowed construction of an 89.6-derived reporter that expressed wild-type *nef* (89.6 VT3; Figure 6A). Consistent with our hypothesis, we found that 89.6 VT3 had reduced LTR activity as assessed flow cytometrically by mCherry expression and 90-fold less *tat/rev* mRNA as assessed by RT-qPCR relative to 89.6 VT1 in transfected HEK293T cells (Figure 6, B and C, respectively). Cotransfection of Tat expressed on a separate plasmid increased LTR activity and partially restored *tat/rev* expression levels (Figure 6, B and C, respectively). Because this effect was partial, we tested cotransfection of *rev*, which also increased mCherry expression in VT3 relative to VT1 in HEK293T cells (Supplemental Figure 8, A–D). Thus, both Tat and Rev limit mCherry expression in VT3. Consistent with the extremely low *tat/rev* expression in 89.6 VT3, CEM-SS cells inoculated with it and treated with PMA and ionomycin were resistant to latency reversal (Figure 6D) as evidenced by the minimal increase in mCherry MFI in infected cells. These data support the conclusion that the presence of a constitutive promoter between the *tat/rev* exons and the resulting larger intron size in 89.6 VT3 relative to 89.6 VT1 lowered *tat/rev* expression, increased latency, and created a barrier to latency reversal.

### Overexpression of HIV tat dramatically reduces the impact of viral inoculum on latency establishment.

**Figure 7:**
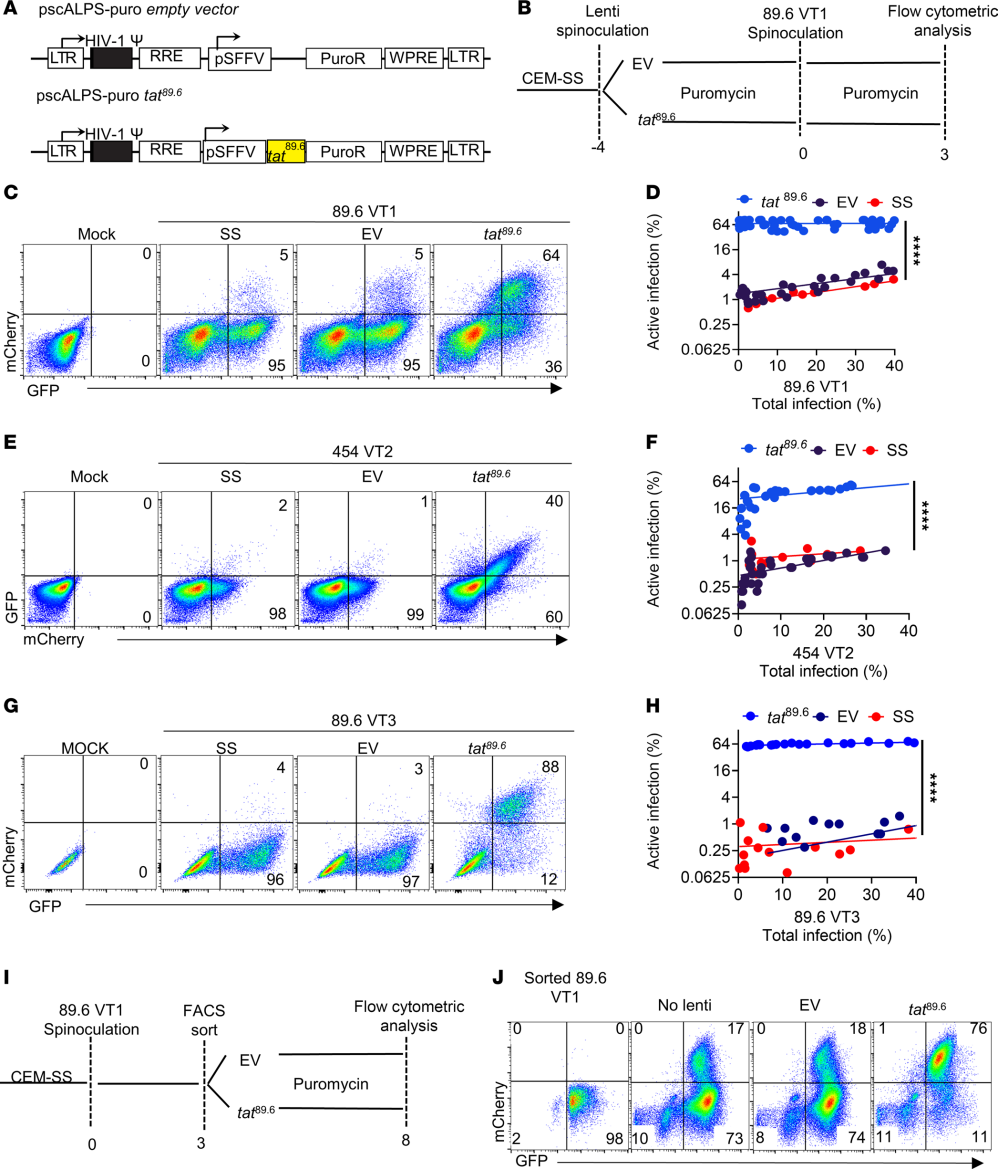
Overexpression of HIV *tat* dramatically reduces the impact of viral inoculum on latency establishment. (**A**) Diagram for lentivirus encoding HIV spliced *tat* and puromycin resistance gene (51). (**B**) Schematic of the experimental process for panels **C**–**H**. (**C**, **E**, and **G**) Representative flow cytometric plots of CEM-SS cells stably expressing lentiviral vectors as indicated and transduced with indicated reporter virus according to the timeline shown in **B**. (**D**, **F**, and **H**) Summary graphs of flow cytometric analysis of CEM-SS cells stably expressing lentiviral vectors as indicated and transduced with increasing amounts of the indicated reporter virus according to the timeline shown in **B**. Each point represents a technical replicate from 3 independent experiments: SS, parental CEM-SS cells; EV, CEM-SS cells stably expressing the empty lentiviral vector; *tat*^89.6^, CEM-SS stably expressing a lentiviral vector containing *tat* as in **A**. (**I**) Schematic demonstrating the experimental process for panel **J**. (**J**) Flow cytometric analysis of CEM-SS cells transduced with 89.6 VT1, sorted for latently infected cells (GFP^+^mCherry^–^), and then transduced with the indicated lentiviral vector according to the timeline shown in **I**. Similar results were obtained in 3 independent experiments. For **D**, **F**, and **H**, statistical significance was determined by Deming (Model II) linear regression, ****P ≤ 0.0001.

Given the inverse correlations we observed between *tat/rev* levels and latency establishment in 89.6 VT1, 454 VT2, and 89.6 VT3, we asked whether Tat was the limiting factor that resulted in latency establishment at low viral inoculum. To test this hypothesis, we utilized a lentiviral vector (51) expressing exogenous *tat* (pscALPS-Tat; Figure 7A). As shown in Figure 7, B–H, utilization of this vector to overexpress *tat*^89.6^ in CEM-SS before infection increased the proportion of active infection for all reporters across a wide range of viral inoculum, dramatically reducing the dependency of active infection on the amount of inoculum added for all constructs. Interestingly, 454 VT2 did not reactivate as completely as 89.6-derived reporters in response to exogenous *tat*, potentially because of the additional latency-promoting effect of the 454 5′ LTR sequences shown in Figure 4. We obtained similar results with respect to Tat overexpression when transduced cells were treated with pscALPS-puro *tat*^89.6^ after HIV integration was established (Figure 7, I and J). Thus, exogenous Tat was sufficient to induce reactivation in CEM-SS cells after viral integration had occurred and latency had been established. In contrast, overexpression of exogenous *rev* in pscALPS did not have a similar effect on reversing latently infected cells (Supplemental Figure 8, E and F, top row).

The impact of *tat* expression on latency was verified utilizing a dual reporter 89.6 VT1 overexpressing *tat*^89.6^ via an internal ribosome entry site (IRES) inserted 3′ to the constitutive SFFV-GFP promoter in 89.6 VT1 (89.6 VT1-IRES-*tat*; Supplemental Figure 9A). Consistent with Tat’s ability to reduce latency in the previous experimental systems, CEM cells inoculated with 89.6 VT1-IRES-*tat*^89.6^ had a reduced proportion of latent versus actively infected cells (Supplemental Figure 9B). In addition, we noted a reduced dependency of active infection on the amount of inoculum when Tat was overexpressed across all viral inoculum tested (Supplemental Figure 9C). Interestingly, however, a subset of cells that overexpressed *tat* maintained low mCherry expression (62%; Supplemental Figure 9B) even at high inoculum (Supplemental Figure 9C), suggesting that *tat* expression from the IRES might be lower than that achieved by pscALPS shown in Figure 7D.

### Lower levels of HIV Tat increase the probability of noninduced provirus in fully activated primary T cells infected in vitro.

**Figure 8:**
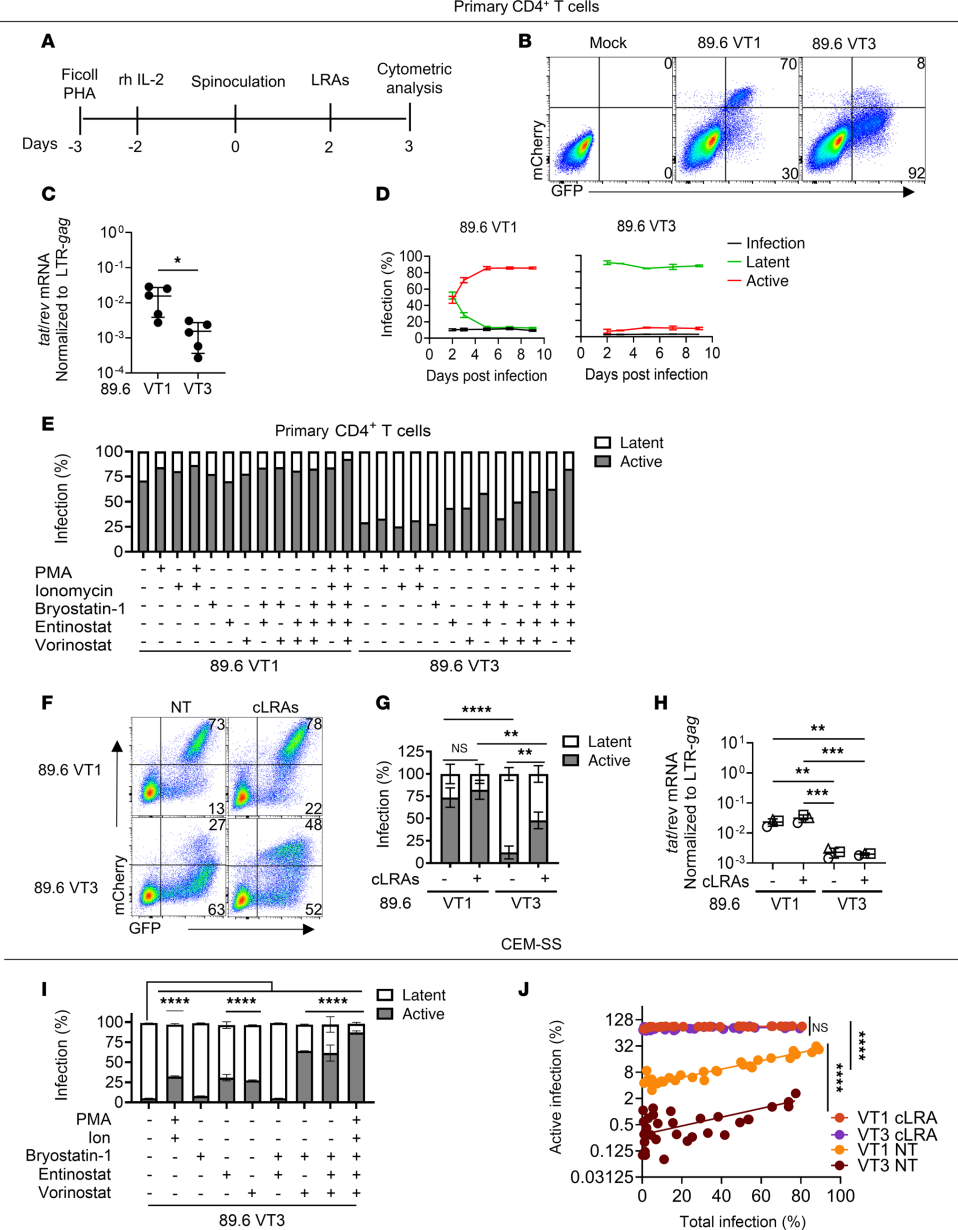
Lower levels of HIV *tat* increase the probability of noninduced provirus in fully activated primary T cells infected in vitro. (**A**) Schematic of the experimental process. (**B**) Flow cytometric analysis of cells transduced with the indicated reporter. (**C**) Summary of RT-qPCR analysis of RNA isolated from cells transduced with the indicated reporter virus as in **A**. *P ≤ 0.05 by 2-tailed unpaired *t* test, *n* = 5. (**D**) Summary of flow cytometric analysis of cells transduced with the indicated reporter virus as in **A** and harvested at the indicated day after infection. *n* = 3. (**E**) Summary graph of flow cytometric analysis of cells treated as in **A**. *n* = 1. (**F**) Flow cytometric analysis of cells treated as in **A** with all 5 LRAs (cLRA). (**G**) Summary graph of flow cytometric data from cells as shown in **F**. *n* = 3. (**H**) Summary graph of RT-qPCR analysis of RNA isolated from cells transduced with the indicated reporter virus and treated where indicated with cLRAs as in **A**. *n* = 3. (**I**) Summary graph of flow cytometric analysis of transduced CEM-SS cells treated with the indicated LRAs. *n* = 3. (**J**) Summary graph of flow cytometric analysis of CEM-SS transduced with increasing amounts of the indicated reporter virus plus or minus cLRAs as indicated in **I**. Statistical significance was determined by Deming (Model II) linear regression. Each point represents a technical replicate from 4 independent experiments. (cLRA included PMA, ionomycin, bryostatin-1, entinostat, and vorinostat.) (NT, no LRA treatment) (89.6 VT1 [VT1], 89.6 VT3 [VT3]). For **C**, **D**, and **G**–**I**, the mean ± standard deviation is shown. For **G**–**I** **P ≤ 0.01; ***P ≤ 0.001; ****P ≤ 0.0001 by 1-way ANOVA with Tukey’s multiple comparisons test.

It is well established that fully activated primary T cells are unlikely to support HIV latency and that latently infected primary T cells isolated from patients can be reactivated through T cell activation (52). However, a barrier to curing HIV is that a proportion of replication-competent HIV proviruses cannot be induced even with maximal T cell activation (53). The explanation for why some integrated fully intact proviral genomes fail to be induced with T cell activation is not well understood. To determine whether Tat levels influence the extent to which latency is established or reversed in these cells, we transduced phytohemagglutinin-activated (PHA-activated) primary CD4^+^ T cells with 89.6 VT1 and 89.6 VT3 according to the timeline shown in Figure 8A. Despite achieving a high level of activation based on markers of T cell activation (Supplemental Figure 10), at 3 dpi, far more 89.6 VT1–transduced cells were actively infected compared with cells transduced with 89.6 VT3 (70% versus 8% of the transduced [GFP^+^] cells; Figure 8B). Consistent with this, *tat/rev* mRNA levels were correspondingly reduced for 89.6 VT3–inoculated primary T cells compared with those treated with 89.6 VT1 (Figure 8C). In addition, most of the cells that initially appeared to harbor latent 89.6 VT1 infection rapidly converted to active infection within about 5 days after infection (Figure 8D). In contrast, activated primary T cells expressing 89.6 VT3 maintained stable latency in approximately 80% of the infected cells for up to 9 dpi (Figure 8D). To determine whether it was possible to induce active infection in primary T cells transduced with 89.6 VT3, we screened a variety of LRAs 2 dpi. We found that 89.6 VT3 was more difficult to reactivate with most LRA combinations as compared with 89.6 VT1. However, when all the LRAs were added in combination (cLRA) (PMA, ionomycin, HDACis [vorinostat and entinostat], and a PKC agonist [bryostatin-1]), we achieved more similar degrees of latency reversal between 89.6 VT1 and 89.6 VT3 despite the differences in *tat/rev* expression (Figure 8E). This was verified in subsequent replicate experiments that demonstrated a strong and significant impact of cLRA treatment on VT3-transduced cells (Figure 8, F and G). However, differences in the extent to which latency could be reversed between 89.6 VT1 and 89.6 VT3 remained (Figure 8G). The mechanism by which cLRA reactivated HIV gene expression was not mediated by induction of higher *tat/rev* levels as the abundance of *tat/rev* was not affected by the addition of LRAs we tested (Figure 8H). Thus, Tat deficiency can cause resistance to LRAs, and low *tat/rev* expression is one possible explanation for proviral genomes that fail to induce with primary T cell activation and some combinations of LRAs. Moreover, many latently infected cells can be reactivated if given the right stimulation even when Tat expression remains low.

In CEM-SS cells, we similarly found that 89.6 VT3 was resistant to most LRA combinations but could be reactivated by the same cLRA cocktail (Figure 8I) albeit to a lesser extent than for VT1 (Supplemental Figure 8F). Interestingly, the addition of exogenous *rev* to the cLRA-treated cells reduced much of this difference (Supplemental Figure 8F), consistent with the *rev* defect we noted in this construct. In addition, this cocktail functioned similarly to overexpression of exogenous *tat* in that it dramatically reduced the effect of inoculum size on active infection in the CEM-SS model for both reporters (Figure 8J). Thus, Tat deficiency and the impact of virus inoculum can be overcome in CEM-SS cells by combinations of LRAs that stimulate T cell activation and histone acetylation pathways.

### Mutation of the TAR element phenocopies low tat levels and creates a barrier to induction of active infection.

**Figure 9:**
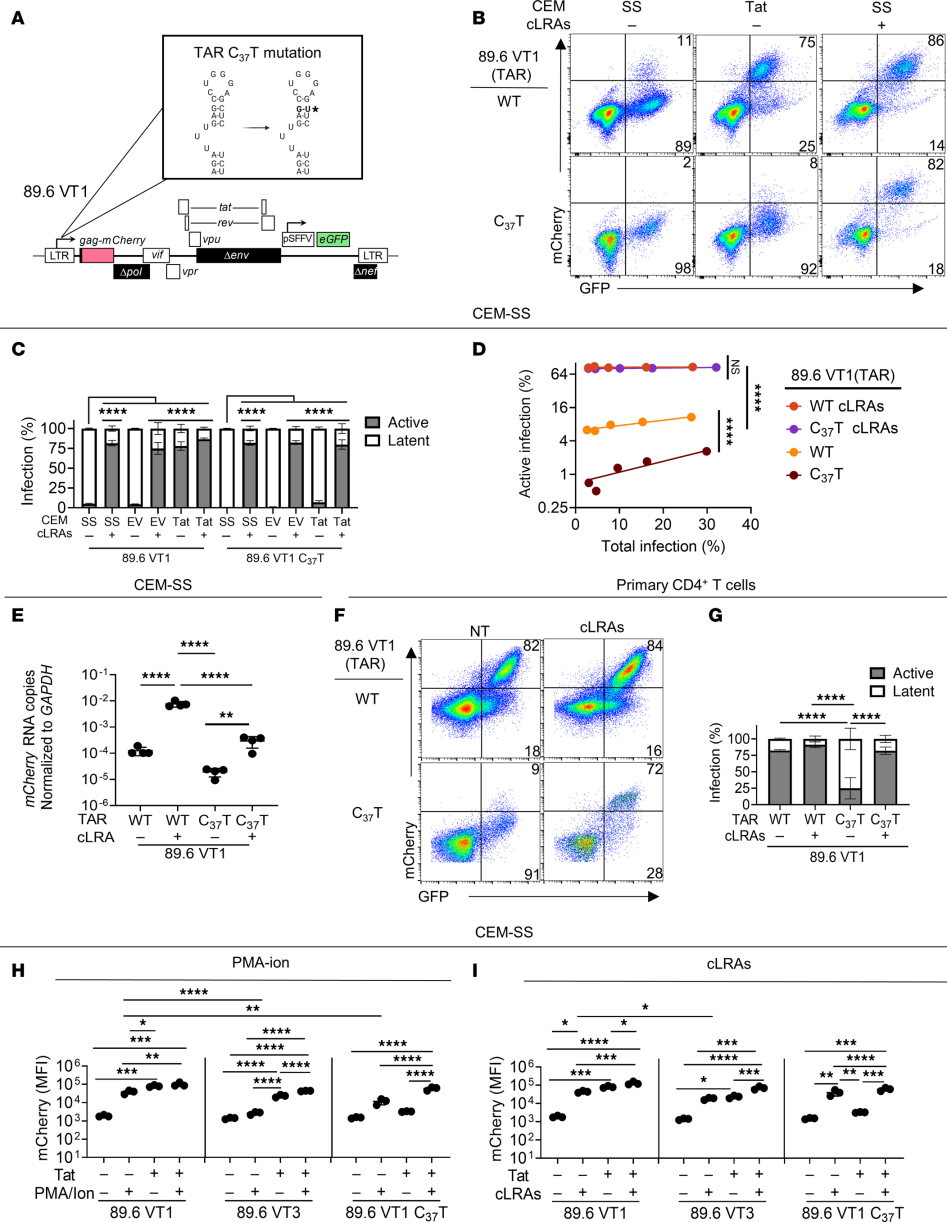
TAR mutation phenocopies low *tat* levels and creates a barrier to induction of active infection. (**A**) Diagram of TAR mutant dual reporter (58). (**B**) Flow cytometric analysis of transduced CEM-SS cells treated with cLRAs (2 dpi) and harvested 3 dpi. Where indicated cells expressed pscALPS-puro *tat*^89.6^ (Figure 7). (**C**) Summary of flow cytometric analysis of cells treated as for **B**. *n* = 3. (**D**) Summary of flow cytometric analysis (as in **B**) with increases in the indicated reporter virus. Active infection was assessed by the proportion of GFP^+^ cells that were also mCherry^+^. Statistical significance was determined by Deming (Model II) linear regression. Each point represents a technical replicate from 1 experiment, *n* = 4. (**E**) Summary graph of RT-qPCR analysis of RNA from transduced CEM-SS treated with cLRAs as for **B**. Untreated cells were sorted for GFP^+^mCherry^–^ whereas cLRA-treated cells were sorted for GFP^+^mCherry^+^ cells 3 dpi. *n* = 4. (**F**) Flow cytometric analysis of transduced PHA-activated primary CD4^+^ T cells treated according to the timeline shown in Figure 8A. (**G**) Summary graph of flow cytometric data from cells as shown in **F**. *n* = 4. (**H** and **I**) Summary graphs of flow cytometric analysis of cells transduced with the indicated reporter construct and treated with PMA and ionomycin (**H**) or cLRA (**I**) as for **B**. *n* = 3. (cLRA included PMA, ionomycin, bryostatin-1, entinostat, and vorinostat.) For **C** and **G**–**I** statistical significance was determined by 1-way ANOVA with Tukey’s multiple comparisons test. The mean ± standard deviation is shown. *P ≤ 0.05; **P ≤ 0.01; ***P ≤ 0.001; ****P ≤ 0.0001.

Tat binding to the TAR element is necessary for maximal Tat transactivation activity. However, some activities of Tat reported to activate signal transduction and transcription factor activation pathways may occur in a manner that is TAR independent (26–30, 54–57). To determine whether the activity of Tat we observed depended on its interaction with the TAR element, we generated a version of 89.6 VT1 that harbored a mutation (C_37_T) in the DNA sequence encoding the TAR element (Figure 9A). Prior studies have shown this mutation reduces Tat-TAR affinity and favors latency establishment (58). In transiently transfected HEK293T cells, the C_37_T mutation resulted in a relatively small but statistically significant reduction in LTR activity, which was abolished with TNF-α treatment (Supplemental Figure 11, A and B), as previously reported (58). Also similar to previous studies (58), mutation of the TAR element dramatically reduced active infection in untreated T cell lines (Figure 9, B–D). In addition, and as expected, mutation of the TAR element abrogated reactivation by exogenous *tat* (Figure 9, B and C).

We also found that mutation of the TAR resembled low Tat expression (89.6 VT3) in the following ways: (i) latency was reduced with cLRA, eliminating differences between wild-type and Tat/TAR-deficient constructs in CEM-SS cells (Figure 9, B–D); (ii) RNA expression with LRA treatment was lower than for the parental construct (89.6 VT1) even after cLRA treatment (Figure 9E); and (iii) there was a high rate of latency in activated primary T cells that could be reduced with cLRA (Figure 9, F and G). The TAR mutation differed from the low-Tat condition (89.6 VT3) in that cLRA treatment was able to fully overcome the TAR deficiency in activated primary T cells (Figure 9, D and G), whereas when Tat levels were low, cLRA did not fully reverse latency in this cell type (Figure 8G).

In addition to examining the proportion of actively infected cells based on a threshold level of mCherry defined by our flow cytometry gates (mCherry^+^ cells), our reporter constructs enable an analysis of the relative per-cell amount of viral gene expression as assessed by mCherry MFI, which allows additional resolution. Therefore, we compared the effect of combinations of LRAs plus or minus exogenous Tat on relative per-cell levels of mCherry (MFI) under conditions of Tat sufficiency (89.6 VT1), Tat deficiency (89.6 VT3), and TAR mutation (89.6 VT1 C_37_T) (Figure 9, H and I, and Supplemental Figure 11C). As described above for assessments quantifying the proportion of mCherry^+^ cells, there were many similarities in LRA responsiveness for the Tat-deficient and TAR-mutant reporter constructs. In addition, we again observed that mutation of the TAR element dramatically decreased responsiveness to exogenous *tat* as assessed by mCherry MFI in the absence of LRAs (Figure 9, H and I, and Supplemental Figure 11C). Moreover, for reporters with an intact TAR element (89.6 VT1 and 89.6 VT3), we again observed that exogenous *tat* and cLRA were similarly effective at reversing latency. Unexpectedly, we observed that in the presence of suboptimal LRA treatment (e.g., PMA and ionomycin alone), exogenous *tat* significantly increased mCherry MFI even when the TAR element was mutated (Figure 9H and Supplemental Figure 11C). The capacity of exogenous *tat* to augment the effect of LRAs in this setting could result from TAR-independent effects of Tat on signal transduction pathways reported by others (26–30, 54–57).

## Discussion

A better understanding of the mechanisms that govern latency establishment and reversal is needed to eradicate latently infected cells. Here, we introduce 3 dual reporters (89.6 VT1, 89.6 VT3, and 454 VT2) and show they establish bona fide, reversible latent infection in the CEM-SS T cell line, HSPCs, and primary CD4^+^ T cells. Similar to other latency reporter viruses (33–37) studied in the Jurkat cell line model, we found that in CEM-SS cells all our reporters established transcriptional latency immediately upon integration and that latency was reversible with a panel of LRAs. For all reporters tested, we observed very few cells (<0.001%) that expressed only the LTR reporter, indicating that deletion because of homologous recombination was not occurring at a high frequency despite a small region of homology at the C- and N-termini (59). Also similar to other studies (60), the latency reporter we described identified populations of activated primary CD4^+^ T cells that appeared to be latently infected. We extended prior findings to show that apparent latency establishment in activated primary T cells was transient under conditions of relatively high *tat* expression and only persisted longer than 5 days in cells harboring genomes that were *tat* deficient. Thus high *tat* levels increased the likelihood of active infection occurring at later time points after integration, even as activated cells began to become quiescent.

In comparing the panel of viral reporters over a range of viral inoculum, we were impressed by the extent to which the proportion of active infection depended on the viral inoculum and the rate of total infection as determined by the constitutive reporter gene. Moreover, we verified this finding with wild-type virus. The magnitude of the effect of inoculum size we observed was quite dramatic and suggests that in vivo, latency is more likely to occur when viral levels are low, such as occurs at the start of a new infection. Conversely, active infection would be favored when the multiplicity of infection was high, as in untreated infection or cell-to-cell spread of virus across virological synapse (61, 62). Preference for latent infection with low titers of virus may reflect an advantage to the virus to establish latency under these conditions. For example, early in infection prior to establishment of viremia, it may be advantageous for the newly infected cell to avoid inhibitory effects of the innate immune response. Alternatively, it may benefit the virus to establish latency in a quiescent T cell, extending its survival time and then reemerging upon T cell activation, when there may be more opportunities for spread. Latency, once established, provides an opportunity for the virus to reappear at a more favorable time.

Interestingly, the dependence of active versus latent infection on inoculum size was abolished by activation of the cells with LRAs. Moreover, the extent to which active infection depended on inoculum size varied among the viral constructs tested. This combination of results suggests that a viral factor behaving similarly to PMA and ionomycin might contribute to the likelihood that infected cells establish latent versus active infection.

Our studies did not reveal effects of Vpr or Nef on latency establishment in short-term culture of CEM-SS cells. A lack of effect of Nef on HIV latency is consistent with a prior report (63). Nevertheless, it remains possible that other *vpr* or *nef* alleles or other cell systems could reveal a phenotype. For example, prior reports found that extracellular Vpr in plasma can reverse latency (64–66). In addition, Vpr has been shown to mediate an effect on clonal populations whereby major *vpr*^+^ clones contained fewer LTR-active cells than *vpr*^–^ clones, presumably due to the cytopathic effect of Vpr on actively infected cells (67).

Similar to other studies, we observed that naturally occurring LTR sequence variations influenced the likelihood of latency establishment (36). A limitation in the interpretation of the latter studies is that the chimeric virus containing only the 5′ LTR region of patient-derived isolates may not include compensatory mutations elsewhere in the genome. Additionally, the cloning strategy resulted in the carryover of a small segment (89 bp) of NL4-3 LTR sequence within all the chimeric viruses. Nevertheless, the fact that 454 VT2 was less responsive to exogenous *tat* than 89.6-derived reporters supports the possibility that sequence differences unrelated to *tat*, such as those in 454 LTR, can affect latency.

The relatively small but significant effect of LTR variation we observed was not sufficient to explain the more substantial effect of inoculum size. In contrast, additional studies revealed striking differences in *tat/rev* expression among reporters that correlated inversely with latency establishment. We found that differences in *tat/rev* expression resulted at least in part from the positioning of the constitutive promoter between the *tat/rev* exons. This conclusion is based on the fact that *tat/rev* levels were relatively high when the promoter was inserted in the *nef* open reading frame (89.6 VT1) and in unmodified genomes (89.6 and 454) as compared with when a promoter was inserted between the *tat/rev* exons (89.6 VT3 and 454 VT2). The observation that *tat* levels were an important determinant for the establishment of viral latency is consistent with prior studies showing that mutations in Tat or its binding element (TAR) promote latency (50, 58, 68, 69). They are also consistent with studies showing that exogenous addition of Tat can reverse latency (63, 70) and that fluctuations in *tat* levels influence the latency decision (71). Moreover, there is extensive sequence diversity within Tat that can affect its function (for review see ref. 72). However, the extent to which variation in sequence alters *tat* levels sufficiently to be a determinant of latency in vivo will require further validation.

While it was not surprising that variations in *tat* levels impacted HIV gene expression, utilization of latency reporters allowed the quantitative comparison of its efficiency compared with other latency reversal strategies. These results revealed the impressive extent to which overexpression of Tat reversed latency establishment, mirroring the effects of maximal T cell activation with LRAs. In addition, we found that exogenous expression of Tat could reverse latency regardless of whether it was introduced before or after integration. This result differs from a prior study showing that preexisting Tat can inhibit active infection (73). In addition, we observed that cells infected with the TAR mutant construct were resistant to latency reversal by both PMA and ionomycin as well as exogenous Tat. Thus, most of the effect of Tat we observed was mediated through its interaction with TAR. However, we did observe an effect of exogenous *tat* in cells harboring the TAR mutant in the setting of suboptimal LRA stimulation, supporting the possibility that Tat can stimulate signal transduction or histone acetylation pathways (26–30, 54–57) in a TAR-independent manner that could have more a global effect on cellular transcriptomic pathways (74). While the precise mechanism requires additional studies, there is evidence based on modeling studies that Tat can act as a positive feedback circuit to regulate latency independent of cell activation (75). Moreover, having a hardwired circuit that supports latency may be evolutionarily important in enhancing virus transmission (76).

Consistent with the known function of Rev to bind the RRE within incompletely spliced RNAs and promote their export to the cytoplasm, we found that Rev deficiency limited the expression of mCherry, which is translated from unspliced mRNA and contains an RRE. Unlike Tat, expression of exogenous Rev in the CEM latency model system did not reverse latency, but rather affected the degree to which viral and reporter proteins made from unspliced and partially spliced mRNA were expressed. Although this was most apparent with VT3, lower *rev* transcript levels in VT1 compared with the wild-type virus potentially explain the low Vpr expression from this construct, as Vpr is made from a partially spliced transcript.

In addition, the latency reporter viruses allowed the quantification of responsiveness to LRAs under conditions of different *tat/rev* expression levels. We observed that *tat/rev* expression correlated with responsiveness to LRAs. The mechanism by which Tat augments or synergizes with LRAs is not clear but likely involves its ability to recruit HATs and promote polymerase processivity. While higher levels of Tat made it easier to reverse latency, we found that even profound Tat deficiency could be overcome by increasing the number of LRAs that inhibit histone deacetylation and stimulate transcription factor activation. Thus, the role of Tat appears to be mechanistically limited to stimulation of these pathways. The capacity to fully overcome Tat deficiency with LRAs was more apparent in CEM-SS cells than in activated primary T cells, as residual latently infected cells remained in 89.6 VT3–transduced primary cells even after maximal cLRA treatment. Although prior studies also found that TAR mutations can be overcome by stimulation of cellular activation pathways (58), investigation of the TAR mutant in the context of the reporter virus allowed us to obtain a more detailed understanding of the extent to which virally infected cells remained uninduced with LRA treatment and in the presence of exogenous *tat*.

In sum, our results show that the amount of Tat experienced by cells following infection is an important variable that determines whether latency is established. In addition, we show that *tat* expression determines how easily a latently infected cell can be reactivated from latency. These results plus our demonstration that *tat* levels can vary in a small sample of HIV molecular clones isolated from patients suggests a possible explanation for why some fully intact viral genomes are difficult to reactivate even by maximal T cell activation in patient T cells stimulated ex vivo (53). Because even profound Tat deficiency can be overcome by increasing the number of LRAs targeting cell activation and histone acetylation pathways, our studies also suggest that the mechanism utilized by Tat mirrors that of LRAs. However, restriction of Tat activity to the LTR through the TAR element makes Tat a more selective LRA and supports the development of novel approaches to deliver HIV Tat to infected cells for latency reversal. For example, Tat delivered via exosomes can reverse latency (77), and utilization of a truncated *tat* with fewer TAR-independent effects may be a viable treatment strategy (78–80).

## Methods

Further information may be found in Supplemental Methods.

### Sex as a biological variable.

As the sex of the anonymous human leukocyte donors was not known, sex was not considered as a biological variable.

### Statistics.

All statistical analyses were performed using GraphPad Prism 10 software as described in the figure legends. *P* < 0.05 was considered statistically significant.

### Study approval.

Anonymized leukocytes isolated by apheresis were obtained from New York Blood Center and determined to be exempt from human studies requirements by the University of Michigan Institutional Review Board.

### Data availability.

Supporting data are available in the Supporting Data Values XLS file and from the corresponding author upon request.

## Author contributions

FGR, VHT, CC, MMP, MCV, and MEYM designed research studies, conducted experiments, and acquired data. FGR and KLC wrote the manuscript. VHT, MMP, and MCV edited the manuscript.

## Supplementary Material

Supplemental data

Unedited blot and gel images

Supporting data values

## Version 1 12/05/2024

In-Press Preview

## Version 2 01/23/2025

Electronic publication
