# Supplementary material for: Variation in HIV-1 Tat activity is a key determinant in the establishment of latent infection
Source: JCI Insight. 2024 Dec 5;10(2):e184711. doi: 10.1172/jci.insight.184711 (PMC11790021; doi:10.1172/jci.insight.184711)
Supplement: Supplemental data [file jciinsight-10-184711-s066.pdf]

Supplemental Information

Variation in HIV-1 Tat activity is a key determinant in the establishment of latent infection.

Francisco Gomez-Rivera[1], Valeri H. Terry[2], Cuie Chen[2φ], Mark M. Painter[1#], Maria C. Virgilio[3,4], Marianne E. Yaple-Maresh[2] ,Kathleen L. Collins[1,2,5*]

[1]University of Michigan, Graduate Program in Immunology, Ann Arbor, MI, USA

[2]University of Michigan, Department of Internal Medicine, Ann Arbor, MI, USA

[3]Department of Computational Medicine and Bioinformatics, University of Michigan, Ann Arbor, MI, USA

[4]Cellular and Molecular Biology Program, University of Michigan, Ann Arbor, MI, USA

[5]University of Michigan, Department of Microbiology & Immunology, Ann Arbor, MI.

*Corresponding author

klcollins@med.umich.edu

Supplemental Methods

Cell Culture

All cell cultures were maintained at 37°C in 5% CO2 humidified atmosphere. Virus producer cells (HEK 293T, ATCC, cat CRL-3216(1)) were maintained in D10 medium [DMEM medium (Gibco, cat 11-995-073) supplemented with 100 U/mL penicillin, 100 µg/mL streptomycin, 2 mM glutamine (Pen-Strep-Glutamine, Invitrogen, cat 10378016), 10 mM HEPES (Invitrogen, cat 15630080), 10% fetal bovine serum (FBS, Gibco, cat 26140079), and 56 µg/mL plasmocin (InvivoGen, cat ant-mpt)]. Hematopoietic stem and progenitor cells (HSPCs) were maintained in STIF medium [Stemspan SFEM II medium (StemCell Technologies, cat 09655) supplemented with 100 ng/ml stem cell factor, 100 ng/ml thrombopoietin, 100 ng/ml Flt3 ligand (StemCell Technologies CC110 cytokine cocktail, cat 02697), and 100 ng/ml recombinant insulin-like growth factor binding protein 2 (R&D Systems, cat 674-B2-025)]. All other cells were maintained in R10 medium [RPMI-1640 medium (Gibco, cat 11875093) supplemented as for D10]. Primary T cells were cultured in R10-50 [R10 plus 50 IU/mL recombinant human interleukin-2 (rIL-2, R&D Systems, cat 202IL010)]. CEM-SS (NIH AIDS Reagent Program, ARP-776) and related cell lines were maintained in R10.

A Tat or Rev expressing cell line, CEM-tat or CEM-rev, was created by transducing CEM-SS cells with pscALPS-Tat or pscALPS-Rev virus and selecting for 5 days with R10 medium containing 250 µg/mL of puromycin (Millipore Sigma, cat P8833).

Isolation and culture of primary T cells from healthy donor blood.

Anonymized leukocytes isolated by apheresis were obtained from the New York Blood Center, and peripheral blood mononuclear cells (PBMCs) were isolated by Ficoll-Paque Plus (GE Healthcare, 17144002) density gradient centrifugation according to manufacturer's protocol. PBMCs were incubated in a 150 mm dish in R10 media for 2 hours at 37°C. $CD8^+$ T lymphocytes were depleted with CD8 Dynabeads according to manufacturer's protocol (Invitrogen, cat 11147D), and the remaining cells were incubated at a density of $2x10^6$ cells/mL in R10 medium and stimulated with 10 µg/ml phytohaemagglutinin (PHA-L, Millipore Sigma, cat 431784). 16-24 hours post-PHA activation, cells were cultured in R10-50. Primary CD4+ T cells were infected via spinoculation at 1090 x *g* for 2 hours at room temperature or treated for other experiments 48 hours after rIL-2 addition.

Isolation and culture of HSPCs from human cord blood.

HSPCs were isolated from whole umbilical cord blood (New York Blood Center) as previously described(2) . Briefly, cord blood mononuclear cells (CBMCs) were isolated from cord blood by Ficoll-Paque (Cytiva, cat 17144002) density gradient centrifugation and either frozen in bovine serum albumin (BSA) (7.5% in phosphate-buffered saline [PBS]; Gibco, cat 10010-023) and dimethyl sulfoxide (DMSO) (10%; Sigma-Aldrich HybriMax, cat D2650) in liquid nitrogen or used immediately. CBMCs were incubated in a 150 mm dish in Stemspan II medium for 2 h at 37°C. CD133+ cells were purified from nonadherent cells by magnetic sorting using a CD133 MicroBead kit (Miltenyi Biotech, cat 130-100-830) according to the manufacturer's protocol, with the modification that 1.5 times the recommended ratio of beads to cells was added to increase the purity of the sort. Purity following the magnetic sort was >92% CD133+ cells, which were also positive for CD34 expression. Purified HSPCs were then maintained in STIF medium. HSPCs were expanded for 4 days and then infected via spinoculation.

Dual color latency probe 89.6 VT1 cloning.

HIV-1 89.6 based dual color latency probe, 89.6 VT1, was constructed by digesting 89.6 ΔN-SFG with BsaBI (New England Biolabs, cat R0537S) and StuI (New England Biolabs, cat R0187S) and religating after removing the 707 bp *env* fragment to create 89.6 ΔEΔN-SFG. mCherry was inserted into the Gag reading frame by synthesizing a fragment containing the vector AatII (New England

Biolabs, cat R0117S) site upstream of the 5' LTR through packaging stem loop 4 and adding the mCherry ORF (Clontech, cat 632522) from amino acid 2 in frame with *gag* (Genscript). The synthetic insert contained a PshAI (New England Biolabs, cat R0593S) site immediately after the mCherry stop codon. 89.6 ΔEΔN-SFG and the insert were digested with AatII and PshAI and ligated to form the final 89.6 VT1 dual color reporter virus. For 89.6 VT1 Δ*vpr*, deletion of *vpr* was achieved using Q5 Site-Directed Mutagenesis kit (New England Biolabs, cat E0554S) where the majority of the *vpr* coding sequence was deleted using PCR exclusion (forward – 5'-CAGAATTGGGTGTCGACATAG-3', reverse – 5'-TCACAGCTTCATTCTTAAGC-3') following the manufacturer's instructions. Primers were designed using the NEB Base Changer online software for base exclusion (https://nebasechanger.neb.com/). To make 89.6 VT3, a 1768 bp PCR fragment containing the vector AatII site, mCherry and PshAI site in *pol* and a 1480 bp fragment containing the StuI site, pSFFV-EGFP and *env* BsaBI site were amplified from 89.6 VT1 with 15 bp homology to the neighboring fragment at each end. 89.6 plasmid (NIH AIDS Reagent Program #3552) was digested with the combination of AatII, PshAI, StuI and BsaBI and the 2423 bp PshAI-StuI fragment (3' end of *pol*, *vif*, *vpr*, *vpu* and *tat*/*rev* exon 1) and 4344 bp BsaBI-AatII fragment (3' half of *env*, *nef*, 3' LTR and vector) were gel purified and assembled with the PCR products with the GeneArt Seamless Cloning Enzyme Mix (Invitrogen, cat A14606)

89.6 VT1-IRES was generated by synthesizing an IRES-MCS IDT gBlock fragment (5'-AAATCTAGACCCCTCTCCCTCCCCCCCCCCTAACGTTACTGGCCGAAGCCGCTTGGAATAAGGC
CGGTGTGCGTTTGTCTATATGTTATTTTCCACCATATTGCCGTCTTTTGGCAATGTGAGGGCCCG
GAAACCTGGCCCTGTCTTCTTGACGAGCATTCCTAGGGGTCTTTCCCCTCTCGCCAAAGGAATG
CAAGGTCTGTTGAATGTCGTGAAGGAAGCAGTTCCTCTGGAAGCTTCTTGAAGACAAACAACGT
CTGTAGCGACCCTTTGCAGGCAGCGGAACCCCCCACCTGGCGACAGGTGCCTCTGCGGCCAAA
AGCCACGTGTATAAGATACACCTGCAAAGGCGGCACAACCCCAGTGCCACGTTGTGAGTTGGAT
AGTTGTGGAAAGAGTCAAATGGCTCTCCTCAAGCGTATTCAACAAGGGGCTGAAGGATGCCCAG
AAGGTACCCCATTGTATGGGATCTGATCTGGGGCCTCGGTGCACATGCTTTACATGTGTTTAGTC
GAGGTTAAAAAAACGTCTAGGCCCCCCGAACCACGGGGACGTGGTTTTCCTTTGAAAAACACGA
TGATAATATGGCCACAACCACCGGTCAGGTTAACATCGATACGCGTGTTTAAACAAA-3') and cloning it into 89.6 VT1 using the XbaI (New England Biolabs, cat R0145) and PmeI (New England Biolabs, cat R0560) sites. 89.6 VT1-IRES-*tat* was generated by synthesizing a *tat* IDT g-block fragment, AgeI-Tat-PmeI, (5'-AAAACCGGTATGGAGCCAGTAAATCCTAGCCTAGAGCCCTGGAAGCATCCAGGAAGTCAGCCTA
AAACTGCTTGTACCAATTGCTATTGCAAAAAATGTTGCTTTCATTGCCAAGCTTGTTTCATAACAA
AAGGCTTAGGCATCTCCTATGGCAGGAAGAAGCGGAGACAGCGACGAAGACCTCCTCAAGACA
GTCAGACTCATCAAGTTTCTCTATCAAAGCAACCCTCCTCCCAGCCTCGAGGGGACCCGACAGG
CCCGAAGGAACAGAAGAAGAAGGTGGAGAGAGAGACAGAGACAGATCCGGTCCATTAGGTTTA
AACAAA-3') and cloning into the AgeI (New England Biolabs, cat R3552) and PmeI sites of 89.6 VT1-IRES.

89.6 VT1 C37T was generated by synthesizing a IDT gBlocks gene fragment (5'-CCACCTGACGTCTAAGAAACCATTATTATCATGACATTAACCTATAAAAATAGGCGTATCACGAG
GCCCTTTCGTCTCGCGCGTTTCGGTGATGACGGTGAAAACCTCTGACACATGCAGCTCCCGGAG
ACGGTCACAGCTTGTCTGTAAGCGGATGCCGGGAGCAGACAAGCCCGTCAGGGCGCGTCAGC
GGGTGTTGGCGGGTGTCGGGGCTGGCTTAACTATGCGGCATCAGAGCAGATTGTACTGAGAGT
GCACCATCTTTAAGACCAATGACTTACAAGGCAGCTGTAGATCTTAGCCACTTTTTAAAAGAAAA
GGGGGGACTGGAAGGGCTAGTTCACTCCCAGAAAAGACAAGATATCCTTGATCTGTGGGTCTAC
CACACACAAGGCTTCTTCCCAGATTGGCAGAACTACACACCAGGGCCAGGAATCAGATATCCAC
TGACCTTTGGATGGTGCTACAAGCTAGTACCAGTTGAGCCAGATGAAGGAGAGAACAACAGAGA
GGACAACAGCTTGCTACACCCTGCTAACCAGCATGGAGTAGAAGACTCGGAGAGACAAGTGTTA
GTGTGGAGGTTTGACAGCCGCCTAGCATTCCATCACGTGGCCCGAGAGCTGCATCCGGAGTAC
TTCAAGAACTGAACTGCTGACACTGAGCTATCTACAAGGGACTTTCCGCTGGGGACTTTCCAGG
GAGGTGTGGCCTGGGCGGAACTGGGGAGTGGCGAGCCCTCAGATGCTGCATATAAGCAGCTG

CTTTTTGCCTGTACTGGGTCTCTCTGGTTAGACCAGATCTGAGCCTGGGAGTTCTCTGGCTAGCT
AGGGAACCCACTGCTTAAGCCTCAATAAAGCTTGCCTTGAGTGCTTCAAGTAGTGTGTGCCCGT
CTGTTGTGTGACTCTGGTAGCTAGAGATCCCTCAGACCATCTTAGTTAGTGTAGAAAATCTCTAG
CAGTGGCGCCCGAACAGGGACCGGAAAGCGAAAGAGAAACCAGAGGAGATCTCTCGACGCAG
GACTCGGCTTGCTGAAGCGCGCACAGCAAGAGGCGAGGGGCGGCGACTGGTGAGTACGCCAA
ATTTTTTGACTAGCGGAGGCTAGAAGGAGAGAGATGGGTGCGAGAGCGTCAGTGAGCAAGGGC
GAGGAGGATAACATGGCCATCATCAAGGAGTTCATGCGCTTCAAGGTGCACATGGAGGGCTCC
GTGAACGGCCACGAGTTCGAGATCGAGGGCGAGGGCGAGGGCCGCCCCTACGAGGGCACCCA
GACCGCCAAGCTGAAGGTGACCAAGGGTGGCCCCCTGCCCTTCGCCTGGGACATCCTGTCCCC
TCAGTTCATGTACGGCTCCAAGGCCTACGTGAAGCACCCCGCCGACATCCCCGACTACTTGAAG
CTGTCCTTCCCCGAGGGCTTCAAGTGGGAGCGCGTGATGAACTTCGAGGACGGCGGCGTGGTG
ACCGTGACCCAGGACTCCTCCCTGCAGGACGGCG -3’) and cloning it into 89.6 VT1 using the AatII and SbfI (New England Biolabs, cat R3642) sites by standard restriction digest and ligation. Underlined sequences refer to modified TAR.

Donor proviral 5’ region cloning into 89.6 VT1.

Donor-derived sequences in the 5’LTR corresponding to HXB2 positions 39 to 806 were amplified out of 2LTR gag GFP constructs with a degenerate LTR primer containing 89.6 sequences flanking the 5’ LTR (3) AccI site and a hybrid reverse primer spanning the second codon of mCherry through the MscI site and *gag*/SL4. The PCR product was digested with AccI (New England Biolabs, cat R0161S) and MscI (New England Biolabs, cat R0534S) and cloned into a shuttle vector created by removing the PshAI-SapI (New England Biolabs, cat R0569) fragment containing HIV-1 *pol* through 3’ LTR then religating the 89.6 VT1 vector. The shuttle vector contained a unique AccI site in the 5’ LTR and a single MscI site in the 5’ end of mCherry. The donor sequence was subsequently cloned into full length 89.6 VT1 by exchanging the AatII-SbfI fragment containing vector backbone, 5’LTR and a portion of the mCherry gene.

454 VT2 cloning.

HIV provirus 454 derived proviral sequence from *pol* to the XhoI site in *nef* (positions 4765-9255 in pHIV454304,(3) was added to pMA-454BX’-gagGFP, an ampicillin-resistant variant of the 2LTR reporter construct p454BX-gagGFP(3) immediately after the EGFP stop codon at the XhoI site. The vector was linearized by XhoI digest (New England Biolabs, cat R0146S) and dephosphorylated with Shrimp Alkaline Phosphatase (New England Biolabs, cat M0371). The insert was prepared by PCR with primers containing 15 bp overlaps with the cut ends of the vector. GeneArt Seamless Cloning and Assembly Enzyme Mix (Invitrogen, cat A14606) was used according to the manufacturer’s instructions to assemble p454-gagGFP. The vector was linearized with a 709 bp deletion in *env* at positions 7203-7911 of pHIV454304 by inverted PCR. An insert containing constitutively expressed mCherry driven by the EF1-α promoter was amplified from positions 3078-4007 of lenti U6-sgRNA/EF1-α-mCherry (4) (Addgene, cat 114199) with primers containing 15 bp homology with the vector ends. The final dual reporter construct was assembled with the GeneArt Seamless Cloning Enzyme Mix. A Nef deficient version of 454 VT2 was created by digesting, filling in and religating the unique XhoI site in *nef*.

Construction of pscALPS-Tat

pscALPS-Tat was generated by cloning the AgeI-Tat-PmeI gBlocks Gene Fragment into the AgeI and PmeI sites of pscALPS (Addgene plasmid 128504)(5).

Construction of pscALPS-Rev

pscALPS-Rev was generated by cloning the BamHI-Rev-NotI gBlocks Gene Fragment into the BamHI and NotI sites of pscALPS (Addgene plasmid 128504).
AAAGGATCCATGGCAGGAAGAAGCGGAGACAGCGACGAAGACCTCCTCAAGACAGTCAGACTC
ATCAAGTTTCTCTATCAAAGCAACCCTCCTCCCAGCCTCGAGGGGACCCGACAGGCCCGAAGGA

ACAGAAGAAGAAGGTGGAGAGAGAGACAGAGACAGATCCGGTCCATTAGTGAACGGATTCTTG GCACTTTTCTGGGTCGATTTGAGGAACCTGTGCCTCTTCCTCTACCACCTCTTGAGAAACTTACT CTTGATTGTAACGAGGATTGTGGAACTTCTGGGACGCAGGGGGTGGGAAGCCCTCAAATATTGG TGGAATCTCCTGCAATATTGGAGCCAGGAACTAAAGAATAGGCGGCCGCAAA.

Virus Production

HEK 293T cells (2.5 million) were plated in a 10 cm dish and adhered overnight. For reporter viruses, the following day, 4 µg of VSV-G plasmid (gift from Nancy Hopkins, Massachusetts Institute of Technology, Cambridge, Massachusetts, USA), 4 µg of pCMV-HIV (gift from S.-J.-K. Yee, City of Hope National Medical Center, Duarte, California, USA), and 4 µg of the viral genome of interest were diluted in 952 µL of 150 mM NaCl. For full length virus 12 µg was utilized and pCMV-HIV and VSV-G were omitted. 48 µL of a 1 mg/mL stock of polyethylenimine (PEI, Polyscience Incorporated, cat 23966-2) was added to the plasmid solution and gently vortexed for 10 seconds. The transfection solution was incubated at room temperature for 15 minutes and 1 mL was added to the plate of cells. The transfection solution was scaled accordingly to facilitate transfection of multiple plates at once. Cells were incubated with the transfection solution for 8 hours. The media was then aspirated and replaced with fresh D10. 48 hours post-transfection, media containing the virus was pelleted to remove cell debris and aliquots were frozen at -80 °C until use. All viruses were titrated with CEM-SS cells to determine volumes of virus required for desired infection rates.

Detection of Vpr in viral particles.

Viral supernatants for western blot analysis were prepared from 89.6 VT1 and 89.6 VT1Δ*vpr* as described above. Viral supernatants for 89.6 (NIH HIV Reagent Program, Division of AIDS, NIAID, NIH: Human Immunodeficiency Virus Type 1 89.6 Infectious Molecular Clone (p89.6), ARP-3552, contributed by Dr. Ronald G. Collman) and 89.6Δ*vpr(6)* by plating 160,000 HEK 293T cells into each of a 6-well plate. After 24 hrs, cells were transfected with 2 µg of viral expression plasmid and 8 µL of PEI per well. 48 hrs post transfection, supernatants were collected and stored at -80°C. For detection of viral particle proteins, 100 µL of viral lysates (prepared as described above) were lysed in 150 µL of Blue Loading Buffer (Cell Signaling Technology, cat 7722), sonicated with a Misonix sonicator (Qsonica, LLC. Newtown, CT), boiled for 10 min at 95°C before loading, and separated by 4-20% SDS-PAGE. Vpr expression was assessed by immunoblot analysis using antibodies to Vpr (1:500, NIH HIV Reagent Program, Division of AIDS, NIAID, NIH: Polyclonal Anti-Human Immunodeficiency Virus Type 1 Vpr Protein, Residues 1 to 50 (antiserum, Rabbit), ARP-11836, contributed by Dr. Jeffrey Kopp) and rabbit secondary (1:5000 [0.2µg/ml], goat anti-rabbit IgG, Invitrogen, cat 65-6120). HIV-Ig was used for detection of Gag p24 (1:1000 [50 µg/ml], NIH HIV Reagent Program, Division of AIDS, NIAID, NIH: Polyclonal Anti-Human Immunodeficiency Virus Immune Globulin, Pooled Inactivated Human Sera, ARP-3957, contributed by NABI and National Heart Lung and Blood Institute (Dr. Luiz Barbosa)) with human secondary (1:10000 [0.1 µg/ml], goat anti-human IgG, ThermoFisher, cat 62-8420).

Detection of Tat by western blot

For western blot analysis of HIV Tat, transfected cells were lysed in 50 µL of Pierce IP lysis buffer (ThermoFisher, cat 87787) supplemented with HALT protease inhibitor (ThermoFisher, cat 78429) and incubated on ice for 5 min. Samples were pipetted up and down every minute. Lysate was clarified by centrifuging at 14,000 x *g* for 10 min at 4 ºC. Supernatant was transferred to a clean tube and a BCA was performed to determine protein concentration. 106 µg of protein for each sample was prepared and 4X Laemmli protein sample buffer (Bio-Rad, cat 1610747) was added to a final concentration of 1X. Samples heated at 85 ºC for 5 min. Samples were loaded on a 4-15% Criterion TGX precast midi protein gel (Bio-Rad, cat 5671083) and ran at 150 V for 1 hr. Protein was transferred to a PVDF membrane (BioRad, cat 1704273) with a Trans-Blot Turbo Transfer System (BioRad, cat 1704150) using the pre-programmed “low molecular weight” transfer method (2.5 Amp, 25 V, 5 min). The membrane was blocked with 5% milk (Fisher, cat NC9121673) in TBST (0.05% Tween-20 (Fisher,

cat BP337-500), 150 mM sodium chloride (NaCl, (Fisher, cat BP358-212), 10 mM Tris(hydroxymethyl)aminomethane (Tris, Invitrogen, cat 15504020), pH 8) for 1 hr at room temperature with rocking. The blot was then probed with an HIV Tat monoclonal antibody (Invitrogen, cat MA171509, clone 7) diluted 1:500 in 5% milk in TBST overnight at 4 ºC with rocking. The following day, the blot was washed 3X with TBST for 5 min per wash and probed with goat anti-mouse IgG-HRP secondary antibody (ThermoFisher, cat 31430) diluted 1:10,000 in 5% milk in TBST for 1 hr at room temperature. The blot was then washed 3X with TBST for 5 min per wash and imaged on a Chemidoc Imaging System (BioRad, cat 12003153). The blot was stripped with OneMinute Plus WB Stripping Buffer (GM Biosciences, cat GM6011) for 2 min at RT then rinsed with TBST. The blot was then probed with a monoclonal GAPDH antibody (Sigma-Aldrich, cat WH0002597M1, clone 3C2) diluted 1:1,000 in 5% milk in TBST overnight at 4 ºC. The blot was washed and probed with the goat anti-mouse IgG-HRP secondary as described above, then imaged with a Chemidoc Imaging System.

HEK 293T Transfections and co-transfections

HEK 293T cells (125,000) were plated in a 12-well plate and adhered overnight. The following day, 500 ng of the HIV molecular clone of interest or/and pcDNA3 spliced Tat plasmid were diluted in 78 µL of 150 mM NaCl . 4 µL of a 1 mg/mL stock of polyethyleneimine (PEI, Polysciences Incorporated, cat 23966-2) was added to the solution and gently vortexed for 10 seconds. The transfection solution was incubated at room temperature for 15 minutes, and 80 µL was added to the plated cells. Cells were incubated with the transfection solution for 8 hours. The media was then aspirated and replaced with fresh D10 or D10 containing TNF-α (R & D Systems, cat 210-TA-005), then cells were harvested 24-48 hours post-transfection.

Viral Transductions

CEM-SS cells were resuspended in virus diluted in D10 to achieve the desired infection rate at a density of 500,000 cells/mL. 500 µL of cells was added to the wells of a 48-well plate (250,000 cells/well). Cells were centrifuged at 1090 x *g* for 2 hours at room temperature. Media was then aspirated and replaced with 500 µL of R10. Cells incubated for 48-72 hours depending on the downstream application. Occasionally, cells were transduced in 6-well plates or 24-well plates. Cells were resuspended in virus as described, and 2 mL or 1 mL was added to the wells (1 million or 500,000 cells/well). Cells were centrifuged as described, and media was aspirated, then replaced with 2 mL or 1 mL of R10.

Primary CD4+ T cells were transduced as described above in a 24-well plate, except 4 µg/mL of polybrene (Millipore Sigma, cat H9268-5G) was added to the virus diluted in D10. After centrifugation, media was aspirated and replaced with 1 mL of R10 supplemented with 50 IU/mL of rIL-2.

Combinatory LRAs treatment

Cells were treated 24 hours prior to harvest and cytometric analysis with 8 µM raltegravir (Selleck Chemicals, cat S2005) along with one or combinations of the following treatment conditions: 10 ng/mL of TNF-α , 50 ng/mL of phorbol 12-myristate 13-acetate (PMA, Sigma, cat P1585-5MG), 500 ng/mL of ionomycin (Sigma, cat I0634-5MG), 5 µM Bryostatin-1 (Sigma-Aldrich, cat B7431), 5 µM Entinostat (Selleck Chemical, cat S1053), 5 mM HMBA (Sigma-Aldrich, cat H4663), 5 µM Vorinostat (Selleck Chemical, cat S1047) all in R10 media or R10 supplemented with 50 IU/mL of rIL-2 (for primary T cells).

Quantitative Polymerase Chain Reaction (qPCR)

Total RNA was extracted from HIV-infected T cells using Direct-zol RNA miniprep kit (Zymo Research, cat R2072) according to manufacturer's protocol . The RNA was reverse transcribed to cDNA using High-Capacity cDNA Reverse Transcription Kit with RNAse inhibitor (ThermoFisher Scientific, cat 4368814). Real-time PCR was performed with the QuantStudio Real-Time PCR 3 systems (Thermo Fisher, cat A28137) using 2X Power SYBR Green master mix (Thermo Fisher, cat

4367659) per manufacturer's instructions. Probe-based assays were performed using TaqMan™ Fast Advanced Master Mix (Thermo Fisher, cat 4444557). The amplification program included an initial denaturation step at 95 °C for 10 min, followed by denaturation at 95 °C for 15s and annealing and extension at 60 °C for 1 min for 45 cycles. Product fluorescence was measured after each extension step. The specificity of SYBR Green assay amplification was evaluated by melting curve analysis. The following primers were used for SYBR Green-based mCherry assay: forward, (5'- AGCAGAGGCTGAAGTTGA -3') and reverse (5'- GATGGTGTAGTCCTCGTTGTG -3'). To determine spliced *tat/rev* copies the following primers and probe were used to detect VT1 and VT3: forward (TatRev FOR1 ,5'- AAAAGGCTTAGGCATCTCCTATGGCA -3'), reverse (TatRev REV1, 5'- TTCTTCTGTTCCTTCGGGCCTGT -3') and probe (TatRev PROBE1, 5'- FAM-CTTCGTCGCTGTCTCCGCTTCTTC-BHQ1-3'). The reverse primer 454 TatRev REV1 (5'- CTTCTTCGATTCCTTCTGGCCTGT –3') was substituted to detect VT2 *tat* and *rev* mRNA. The *tat* mRNA specific qRT-PCR assay consisted of a tat specific forward primer (Tat FOR1, 5'- TAAATCCTAGCCTAGAGCCCT -3'), reverse primer (TatRev REV2, 5'- GAGGAGGGTTGCTTTGATAGAG –3') and probe (TatRev PROBE2, 5'-FAM-ACGAAGACCTCCTCAAGACAGTCAGA BHQ1-3'). The *rev* mRNA specific forward primer (Rev FOR4, 5'- GGGCGGCGACTGCT –3') was used with TatRev REV2 and TatRev PROBE2 to quantify *rev* mRNA copies. *tat* and *rev* transcript specificity was confirmed with synthetic templates for VT1 *tat* (5'-
GTCTCTCTGGTTAGACCAGATCTGAGCCTGGGAGCTCTCTGGCTAGCTAGGGAACCCACTGCTT
AAGCCTCAATAAAGCTTGCCTTGAGTGCTTCAAGTAGTGTGTGCCCGTCTGTTGTGTGACTCTGG
TAGCTAGAGATCCCTCAGACCATCTTAGTTAGTGTAGAAAATCTCTAGCAGTGGCGCCCGAACA
GGGACCGGAAAGCGAAAGAAACCAGAGGAGATCTCTCGACGCAGGACTCGGCTTGCTGAAG
CGCGCACAGCAAGAGGCGAGGGGCGGCGACTGAATTGGGTGTCGACATAGCAGAATAGGCATT
ATTCAACAGAGGAGAACAAGAAATGGAGCCAGTAAATCCTAGCCTAGAGCCCTGGAAGCATCCA
GGAAGTCAGCCTAAAACTGCTTGTACCAATTGCTATTGCAAAAAATGTTGCTTTCATTGCCAAGC
TTGTTTCATAACAAAAGGCTTAGGCATCTCCTATGGCAGGAAGAAGCGGAGACAGCGACGAAGA
CCTCCTCAAGACAGTCAGACTCATCAAGTTTCTCTATCAAAGCAACCCTCCTCCCAGCCTCGAGG
GGACCCGACAGGCCCGAAGGAACAGAAGAAGAAGGTGGAGAGAGAGACAGAGACAGATCCGG
TCCATTAGTGAACGGATTCTTGGCACTTTTCTGGGTCGATTTGAGGAACCTGTGCCTCTTCCTCT
ACCACCTCTTGAGAAACTTACTCTTGATTGTAACGAGGATTGTGGAACTTCTGGGACGCAGGGG
GTGGGAAGCCCTCAAATATTGGTGGAATCTCCTGCAATATTGGAGCCAGGAACTAAAGAATAG –3') and *rev* (5'-
GGGTCTCTCTGGTTAGACCAGATCTGAGCCTGGGAGCTCTCTGGCTAGCTAGGGAACCCACTG
CTTAAGCCTCAATAAAGCTTGCCTTGAGTGCTTCAAGTAGTGTGTGCCCGTCTGTTGTGTGACTC
TGGTAGCTAGAGATCCCTCAGACCATCTTAGTTAGTGTAGAAAATCTCTAGCAGTGGCGCCCGA
ACAGGGACCGGAAAGCGAAAGAAACCAGAGGAGATCTCTCGACGCAGGACTCGGCTTGCTG
AAGCGCGCACAGCAAGAGGCGAGGGGCGGCGACTGCTTGTTTCATAACAAAAGGCTTAGGCAT
CTCCTATGGCAGGAAGAAGCGGAGACAGCGACGAAGACCTCCTCAAGACAGTCAGACTCATCA
AGTTTCTCTATCAAAGCAACCCTCCTCCCAGCCTCGAGGGGACCCGACAGGCCCGAAGGAACA
GAAGAAGAAGGTGGAGAGAGAGACAGAGACAGATCCGGTCCATTAGTGAACGGATTCTTGGCA
CTTTTCTGGGTCGATTTGAGGAACCTGTGCCTCTTCCTCTACCACCTCTTGAGAAACTTACTCTT
GATTGTAACGAGGATTGTGGAACTTCTGGGACGCAGGGGGTGGGAAGCCCTCAAATATTGGTG
GAATCTCCTGCAATATTGGAGCCAGGAACTAAAGAATAG –3'). RT-qPCR Primer assay for *GAPDH* (Qiagen, cat 330001, assay PPH00150F) was used as a normalizer; in some cases, total *LTR-gag* copy number (7) [second-LTR-F-univ forward (5'- GTGTIGAAAATCTCTAGCAGTGGC -3'), Gag R3 reverse (5'- CCTTCTAGCCTCCGCTAGTCAA -3') and Gag probe 2 (5'-FAM CGCTTCAGCAAGCCGAGTCCTGC-BHQ-1-3', Biosearch Technologies, cat DLO-RFB-5)] was used to normalize for infection rate. The *LTR-gag* primers were also applied to genomic DNA. The mRNA abundance of each was determined by the copy numbers of the gene by using a synthetic DNA fragment or p89.6VT1 to generate a standard curve. To determine the fold change, total copies of *mCherry,* and spliced *tat/rev* of each sample were normalized to the total copy number of *GAPDH* or

LTR-gag. Normalization for provirus copies was done with qPCR on genomic DNA using forward primer B-globin-F (5'- CCCTTGGACCCAGAGGTTCT-3'), B-globin-R (5'- CGAGCACTTTCTTGCCATGA-3') and B-globin Probe (5'-FAM- ATCTGTCCACTCCTGATGCTGTTATGGGC-BHQ-1-3', Biosearch Technologies, cat DLO-RFB-5).(8). The probe was modified to include residues 7-35 of the published probe with the noted dye and quencher.

Flow cytometry and fluorescence activated cell sorting (FACS).

Cytometric analysis of CEM-SS, HEK-293T, and primary human cells was performed on an Aurora spectral cytometer (Cytek, cat N7-00002). Cells were harvested and pelleted (800 x *g* for 2 minutes at 4ºC) into a 96-well plate and washed twice with 200 µL of FACS Buffer [1% human serum (Fisher, cat BP2525-100), 2% FBS (Gibco, cat 26140079), 2 mM HEPES (Invitrogen, cat 15630080) and 0.025% sodium azide (NaN_3) (Sigma, cat. S8032-25G) in 1X PBS]. Cell viability was determined with 0.04 µg/mL 4′,6-diamidino-2-phenylindole [DAPI, (Fisher, cat 62248)] dissolved in PBS incubated for 5 min at room temperature followed by two washes with 200 µL FACS Buffer. Cells were fixed with 100 µL of 2% paraformaldehyde (PFA, Fisher, cat 50-980-487) for 30 minutes at room temperature, washed twice with 200 µL of FACS buffer, and resuspended in 125 µL of FACS Buffer for flow cytometric analysis.

Antibodies recognizing CD25 (R-phycoerythrin-Cyanine7 (PE-Cy7) conjugated; BD, cat 25-0259-42, clone BC96), CD69 (PE-Cy7 conjugated; eBiosciences, cat 25-0699-42, clone FN50) and HLA-DR (PE-Cy7 conjugated; eBiosciences,, cat 25-9956-42, clone LN3) were used for surface activation marker staining. Cells were suspended in antibody diluted in FACS Buffer and incubated on ice for 30 min, washed, and fixed with 2% paraformaldehyde in PBS for 30 minutes at room temperature. Cells were gated by forward scatter versus side scatter, doublet exclusion and infected cells were gated by the surface marker staining versus side scatter using unstained and isotype to set the positive gate.

Surface expression of MHC-I HLA-A2 was measured using BB7.2 (9) [University of Michigan Hybridoma Core, (0.5 µg/mL)], incubated for 20 minutes at room temperature followed by two washes with FACS Buffer and incubation with goat anti-mouse IgG2b AlexaFluor 647 [Invitrogen, cat A21242, (1 µg/µL)] for 20 minutes. Cells were gated by forward scatter versus side scatter, and infected cells were gated by the MHC-I staining versus side scatter using unstained and isotype to set the positive gate.

Cells inoculated with wild type HIV were stained for intracellular Gag by fixation with 2% paraformaldehyde for 30 minutes at room temperature, permeabilization with 0.1% Triton X-100 (Themo Fisher Scientific, cat BP151-500) at room temperature for 5 minutes, and staining with FITC-conjugated anti-Gag antibody (Beckman Coulter, , cat 6604665, clone KC57) at room temperature for 20 min. Cells were gated by forward scatter versus side scatter, and infected cells were gated by Gag-FITC versus side scatter using mock-infected cells to set the Gag+ gate.

For FACS, dual color reporter transduced CEM-SS and primary T cells were harvested, counted, and pelleted by centrifugation at 500 x *g* for 5 minutes at room temperature, washed twice with 1X PBS) and resuspended at 10 million cells per mL in MACS Buffer [1X PBS, 0.05% BSA (Life Technologies, cat 501215315), 2 mM ethylenediaminetetraacetic acid [EDTA, (Invitrogen, cat 15575020)]. Cells were sorted by fluorescent protein expression (GFP and/or mCherry) on a Bigfoot Spectral Cell Sorter (Thermo Fisher). Cells were gated by forward scatter versus side scatter, and infected cells were gated by constitutive reporter fluorescence versus LTR reporter fluorescence using mock-infected cells to set the infected positive gate. Cytometric data was analyzed using FlowJo version 10 (BD Life Sciences).

Supplementary Figures and Legends

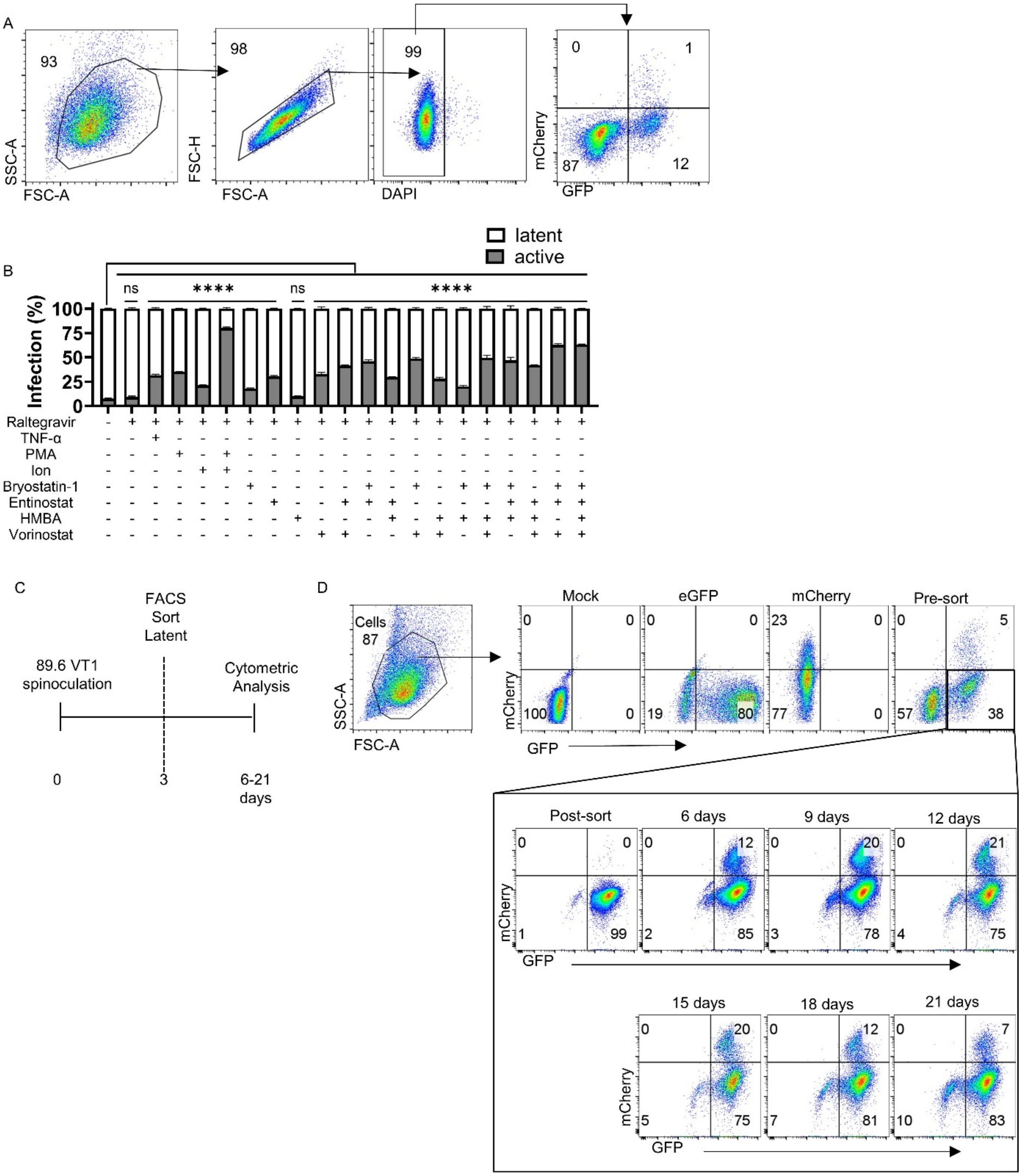

Supplementary Figure 1: 89.6 VT1 latency is stable in CEM-SS and reversible by combination latency reversal agent treatment (LRA). (A) Gating strategy for assessment of latent (GFP⁺, mCherry⁻) or active (GFP⁺, mCherry⁺) infection. **(B)** Summary graph of flow cytometric analysis of CEM-SS treated according to the timeline shown in panel 1B with the indicated LRAs. Statistical significance was determined by Two-way ANOVA with Šídák's multiple comparisons test relative to untreated. ns, not significant; ****P ≤ 0.0001. The mean ± standard deviation is shown for 3 independent experiments. **(C)** Schematic demonstrating the experimental time course. **(D)** Flow cytometric analysis of CEM-SS cells transduced with 89.6 VT1, sorted for GFP⁺ mCherry⁻ (latently infected) and incubated for the indicated time after sorting according to the timeline shown in (C). Similar results were obtained in 3 experiments.

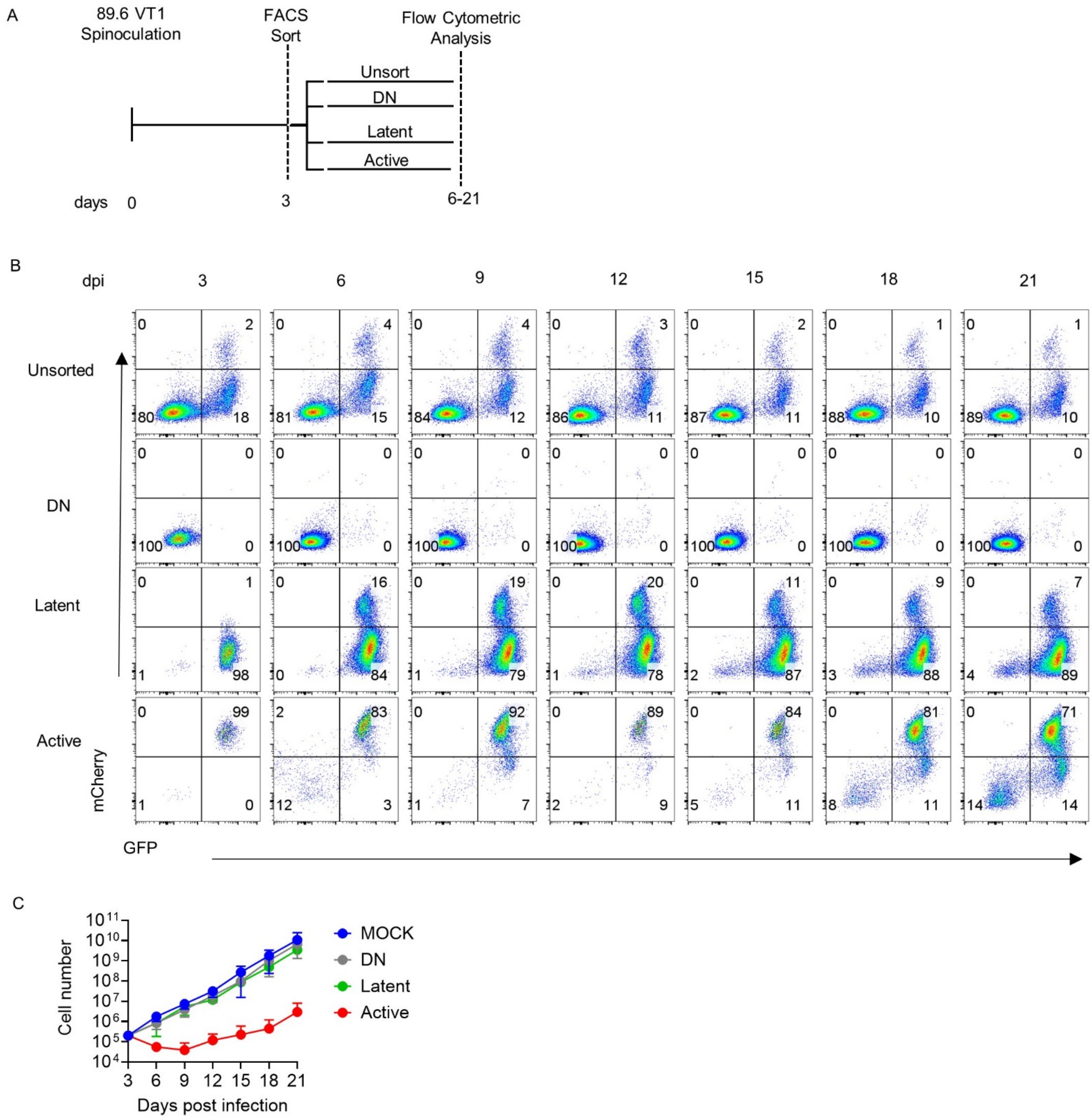

Supplementary Figure 2. 89.6 VT1 actively infected cells can revert to latency and have slower growth kinetics than uninfected cells. **(A)** Schematic demonstrating the experimental time course. **(B)** Flow cytometric analysis of CEM-SS cells transduced with 89.6 VT1, sorted for the indicated cell population and incubated for the indicated time after sorting according to the timeline shown in (A). Similar results were obtained in 3 experiments. **(C)** Summary graph of cumulative cell number over time for the indicated cell population. Results from 3 experiments mean ± standard deviation are shown.

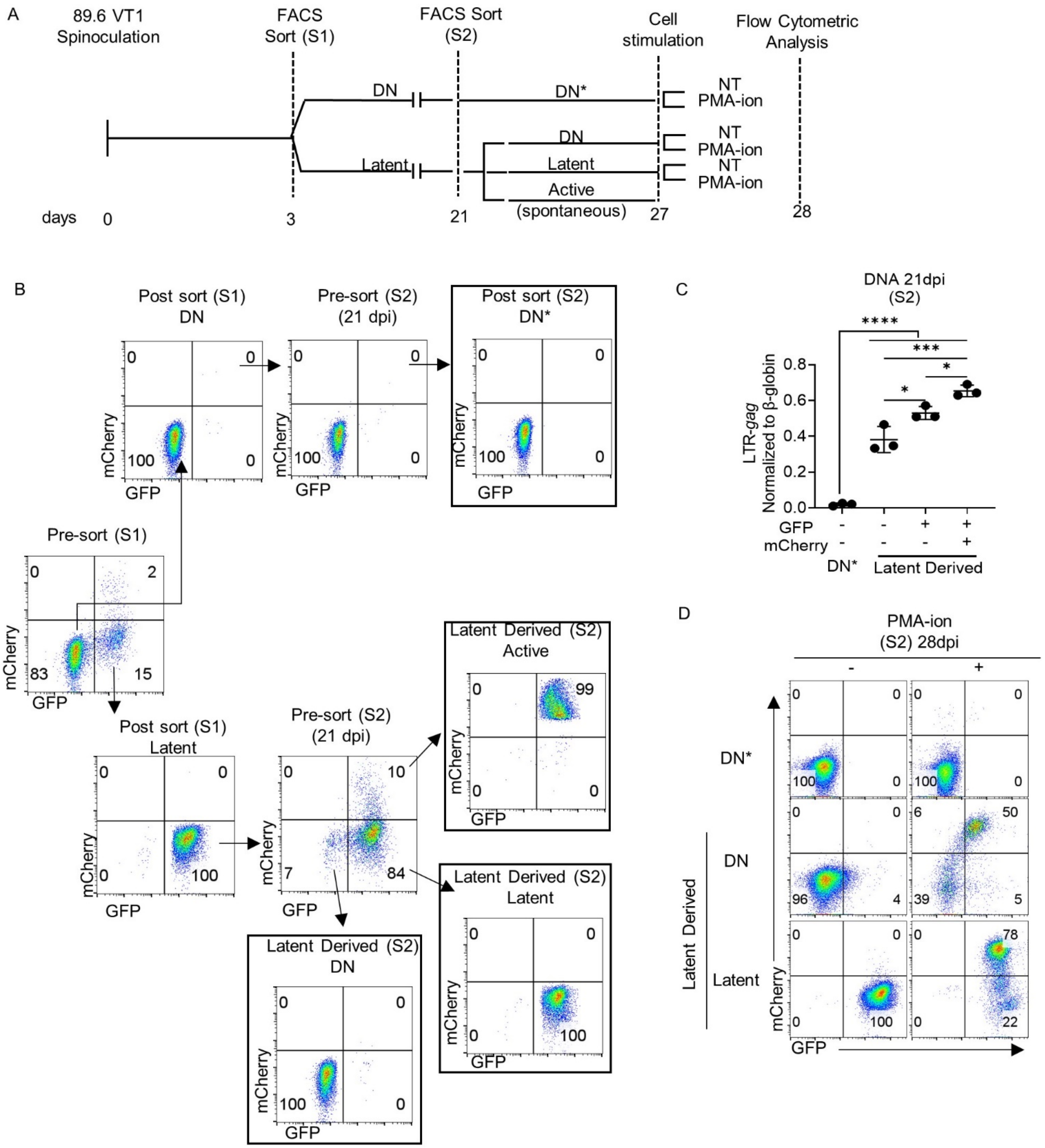

Supplementary Figure 3. 89.6 VT1 latently infected cells can silence fluorescent latency reporter protein expression and become "double negative". (A) Schematic demonstrating the experimental time course. **(B)** Flow cytometric analysis of CEM-SS cells transduced with 89.6 VT1, sorted for the indicated cell population and incubated for the indicated time after sorting according to the timeline shown in (A). Similar results were obtained in 3 experiments. **(C)** Summary graph of -qPCR analysis of RNA isolated from CEM-SS cell populations from part (B). Statistical significance was determined by One-way ANOVA with Tukey's multiple comparisons test. The mean ± standard deviation is shown for 3 independent experiments, *P≤ 0.05,; ***P ≤ 0.001;**** P ≤ 0.0001 . **(D)** Flow cytometic analysis of CEM-SS cells from part C treated with PMA and ionomycin (PMA-ion) where indicated. Similar results were obtained in 3 experiments.

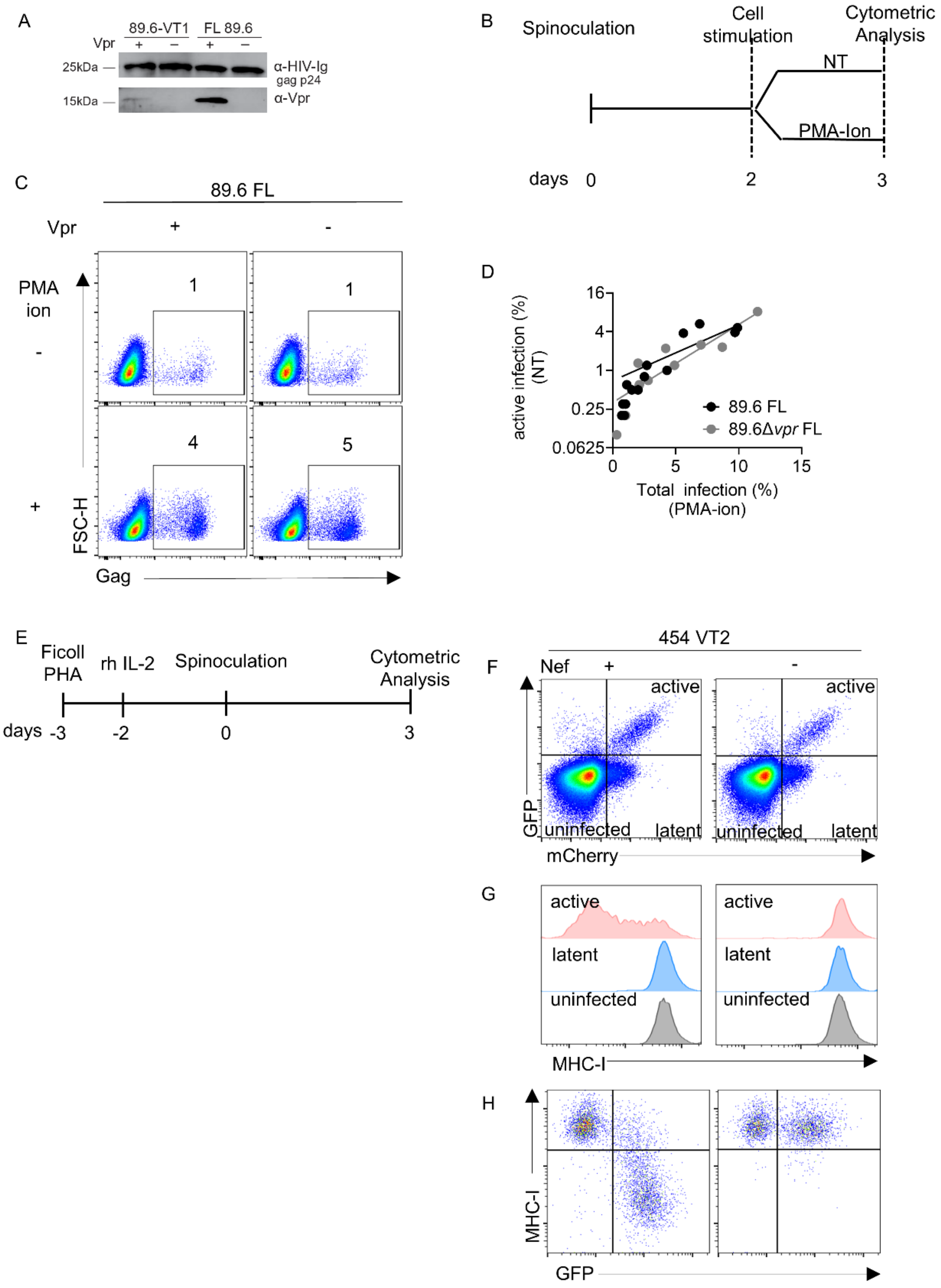

Supplementary Figure 4: Validation of *vpr* and *nef* expression in wild type and mutant 89.6 VT1 and 454 VT2 dual reporters. (A) Representative immunoblot analysis of virus supernatant from HEK-293T cells for the indicated viruses. Similar results were obtained in 2 independent experiments (one comparing VT1 to 89.6 and one to ΔGPE(10) **(B)** Schematic of the experimental process for (C-D). **(C)** Flow cytometric analysis of CEM-SS transduced with the indicated full-length (FL) virus and stained intracellularly for HIV-Gag. Cells were gated from FSC vs SSC, FSC vs. viability and FSC vs HIV-Gag. **(D)** Summary graph of flow cytometric analysis (as in C) of CEM-SS cells transduced with increasing amounts of the indicated FL virus. Active infection was assessed by the proportion of Gag^{+} cells after 24 hours of raltegravir only (NT), and total infection was assessed by the proportion of Gag^{+} cells after 24 hours of PMA-ionomycin (PMA-ion) treatment in the presence of raltegravir. Each dot is a technical

replicate from 1 experiment. **(E)** Schematic of the experimental process for (F-H). **(F)** Flow cytometric analysis of primary $CD4^+$ T cells transduced with the indicated reporter virus. Cells were gated as shown in Supplementary Figure 1. **(G)** Representative flow cytometric analysis for primary $CD4^+$ T cells evaluating MHC-I expression for the populations from panel F. **(H)** Representative flow cytometric plots showing MHC-I downmodulation in GFP^+ cells (active infected) primary $CD4^+$ T cells 3 days post infection with the indicated reporter virus as shown in panel F. Similar results were obtained in 3 experiments.

VT1 5' LTR

										10										20										30
89.6	T	G	G	A	A	G	G	G	C	T	A	G	T	T	C	A	C	T	C	C	C	A	G	A	A	A	A	G	A	C
409	.	.	.	.	.	.	.	.	.	.	.	.	.	.	.	.	.	.	.	.	.	.	.	.	.	.	.	.	.	.
421	.	.	.	.	.	.	.	.	.	.	.	.	.	.	.	.	.	.	.	.	.	.	.	.	.	.	.	.	.	.
439	.	.	.	.	.	.	.	.	.	.	.	.	.	.	.	.	.	.	.	.	.	.	.	.	.	.	.	.	.	.
454	.	.	.	.	.	.	.	.	.	.	.	.	.	.	.	.	.	.	.	.	.	.	.	.	.	.	.	.	.	.
NL4-3	.	.	.	.	.	.	.	.	.	.	.	.	.	.	.	.	.	.	.	.	.	.	.	.	.	.	.	.	.	.

										40										50										60
89.6	A	A	G	A	T	A	T	C	C	T	T	G	A	T	C	T	G	T	G	G	G	T	C	T	A	C	C	A	C	A
409	.	.	.	.	.	.	.	.	.	.	.	.	.	.	.	.	.	.	.	.	.	.	.	.	.	.	.	.	.	.
421	.	.	.	.	.	.	.	.	.	.	.	.	.	.	.	.	.	.	.	.	.	.	.	.	.	.	.	.	.	.
439	.	.	.	.	.	.	.	.	.	.	.	.	.	.	.	.	.	.	.	.	.	.	.	.	.	.	.	.	.	.
454	.	.	.	.	.	.	.	.	.	.	.	.	.	.	.	.	.	.	.	.	.	.	.	.	.	.	.	.	.	.
NL4-3	.	.	.	.	.	.	.	.	.	.	.	.	.	.	.	.	.	.	.	.	.	.	.	.	.	.	.	.	.	.

										70										80										90
89.6	C	A	C	A	A	G	G	C	T	T	C	T	T	C	C	C	A	G	A	T	T	G	G	C	A	G	A	A	C	T
409	.	.	.	.	.	.	.	.	.	.	.	.	.	.	.	.	T	.	.	.	.	.	.	.	.	.	.	.	.	.
421	.	.	.	.	.	.	.	.	.	.	.	.	.	.	.	.	T	.	.	.	.	.	.	.	.	.	.	.	.	.
439	.	.	.	.	.	.	.	.	.	.	.	.	.	.	.	.	T	.	.	.	.	.	.	.	.	.	.	.	.	.
454	.	.	.	.	.	.	.	.	.	.	.	.	.	.	.	.	T	.	.	C	.	.	.	.	.	.	.	.	T	.
NL4-3	.	.	.	.	.	.	.	.	.	.	.	.	.	.	.	.	.	.	.	.	.	.	.	.	.	.	.	.	.	.

										100										110										120
89.6	A	C	A	C	A	C	C	A	G	G	G	C	C	A	G	G	A	A	T	C	A	G	A	T	A	T	C	C	A	C
409	.	.	.	.	.	.	.	.	.	.	.	.	.	.	.	.	G	.	.	.	.	.	.	.	.	.	.	.	C	.
421	.	.	.	.	.	.	.	.	.	.	.	.	.	.	.	.	G	.	.	.	.	.	.	.	.	C	.	.	.	.
439	.	.	.	.	.	.	.	.	.	.	.	.	.	.	.	.	C	.	.	.	.	.	.	.	.	.	.	.	.	T
454	.	.	.	.	.	.	.	.	.	.	.	.	.	.	.	.	G	.	.	.	.	.	.	.	.	.	.	.	.	.
NL4-3	.	.	.	.	.	.	.	.	.	.	.	.	.	.	.	.	G	.	.	.	.	.	.	.	.	.	.	.	.	.

										130										140										150
89.6	T	G	A	C	C	T	T	T	G	G	A	T	G	G	T	G	C	T	A	C	A	A	G	C	T	A	G	T	A	C
409	.	.	.	.	.	.	.	C	.	.	.	.	.	.	.	.	.	.	.	.	.	.	A	.	.	.	.	.	.	.
421	.	.	.	.	.	.	.	.	.	.	.	.	.	.	.	.	.	.	T	.	.	.	.	.	.	.	.	.	.	.
439	.	.	T	G	.	.	.	.	.	.	.	.	.	.	.	.	.	.	T	.	.	.	.	T	.	.	.	.	G	.
454	.	.	.	.	.	.	.	.	.	.	.	.	.	.	.	.	.	.	T	.	.	.	.	.	.	.	.	.	.	.
NL4-3	.	.	.	.	.	.	.	.	.	.	.	.	.	.	.	.	.	.	T	.	.	.	.	T	.	.	.	.	.	.

										160										170										180
89.6	C	A	G	T	T	G	A	G	C	C	A	G	A	T	-	-	-	-	-	-	G	A	A	G	G	A	G	A	G	A
409	.	.	.	.	.	.	.	T	.	.	.	.	.	G	A	A	G	G	T	A	.	.	.	.	A	.	.	.	C	.
421	.	.	.	.	G	.	.	T	A	A	.	.	.	G	C	A	G	G	T	A	.	.	.	.	A	G	.	C	C	.
439	.	.	.	.	.	.	.	T	.	.	.	.	.	.	A	A	G	G	T	A	.	.	.	.	A	G	.	C	C	.
454	.	.	.	.	.	.	.	C	.	.	.	.	.	G	A	A	G	A	T	A	.	.	.	A	A	G	.	C	C	.
NL4-3	.	.	.	.	.	.	.	A	.	.	.	.	.	G	C	A	A	G	T	A	.	.	.	.	A	G	.	C	C	.

										190										200										210
89.6	A	C	A	A	C	A	G	A	G	A	G	G	A	C	A	A	C	A	G	C	T	T	G	C	T	A	C	A	C	C
409	.	T	.	C	A	G	.	.	.	.	A	A	.	.	.	T	.	.	A	.	C	.	.	T	.	.	.	.	.	.
421	.	T	G	.	A	G	.	.	.	.	.	A	.	.	.	.	.	.	.	.	.	.	.	T	.	.	.	.	.	.
439	.	T	C	.	A	G	.	.	.	.	.	A	.	.	.	.	.	.	.	.	C	.	.	T	.	.	.	.	.	.
454	.	T	G	.	A	G	.	.	.	.	A	A	.	.	.	.	.	.	.	.	.	.	.	T	.	.	.	.	.	.
NL4-3	.	T	G	.	A	G	.	.	.	.	.	A	.	.	.	.	.	.	.	.	.	.	.	T	.	.	.	.	.	.

										220										230										240
89.6	C	T	G	C	T	A	A	C	C	A	G	C	A	T	G	G	A	G	T	A	G	A	A	G	A	C	T	C	G	G
409	.	.	A	T	G	.	G	.	.	.	.	.	.	.	.	.	.	A	.	G	.	.	T	.	.	.	C	.	A	.
421	.	C	A	T	A	.	G	.	.	T	.	.	.	.	.	.	G	A	.	G	.	.	G	.	.	T	C	.	.	.
439	.	.	A	T	G	.	G	.	.	.	.	.	.	.	.	.	G	A	.	G	.	.	G	.	.	T	.	.	.	.
454	.	.	A	T	G	.	G	.	.	.	.	.	.	.	.	.	G	A	.	G	.	.	T	.	.	.	C	.	.	.
NL4-3	.	.	A	T	G	.	G	.	.	.	.	.	.	.	.	.	G	A	.	G	.	.	G	.	.	.	C	.	.	.

										250										260										270
89.6	A	G	A	G	A	C	A	A	G	T	G	T	T	A	G	T	G	T	G	G	A	G	G	T	T	T	G	A	C	A
409	.	.	.	A	G	G	.	.	.	.	.	.	.	.	A	.	.	.	.	.	.	A	.	.	.	.	.	.	.	.
421	.	.	G	.	.	G	.	.	.	.	.	.	.	.	.	.	.	.	.	.	.	A	.	.	.	.	.	.	.	.
439	.	.	.	.	.	G	.	.	.	.	.	C	.	.	.	.	A	.	.	.	.	A	.	.	.	.	.	.	.	.
454	.	.	.	A	.	G	.	.	.	.	A	.	.	.	.	A	.	.	.	.	.	A	.	.	.	.	.	.	.	.
NL4-3	.	.	G	.	.	G	.	.	.	.	A	.	.	.	.	.	.	.	.	.	.	A	.	.	.	.	.	.	.	.

Supplementary Figure 5, continued

									280										290										300
89.6	G	C	C	G	C	C	T	A	G	C	A	T	T	C	C	A	T	C	A	C	G	T	G	G	C	C	C	G	A	G
409	.	.	.	T	.	.	.	G	.	.	.	.	A	T	.	G	.	.	.	.	A	.	.	.	.	.	.	.	C	.
421	.	.	.	.	.	.	.	.	.	.	.	.	.	T	A	C	.	.	.	.	A	.	.	.	.	.	.	.	.	.
439	.	.	.	.	.	.	.	.	.	.	.	.	.	T	.	.	.	.	.	.	A	.	.	.	.	.	.	.	.	.
454	.	.	.	A	.	.	.	.	.	.	.	.	.	T	.	G	.	.	.	.	A	.	.	.	.	.	A	A	G	.
NL4-3	.	.	.	T	.	.	.	.	.	.	.	.	.	T	.	G	.	.	.	.	A	.	.	.	.	.	.	.	.	.

									310										320									329	
Region															TCF-1α															
89.6	A	G	C	T	G	C	A	T	C	C	G	G	A	G	T	A	C	T	T	C	A	A	G	A	A	C	T	G	A	-
409	.	.	A	A	.	.	.	.	.	.	.	.	.	.	.	T	T	.	A	.	.	.	.	G	.	.	.	.	C	-
421	.	.	.	.	.	.	.	.	.	.	.	.	.	.	.	.	.	.	A	.	.	.	.	G	.	.	.	.	C	T
439	.	.	.	.	A	.	.	.	.	.	.	.	.	.	.	.	.	.	A	.	.	.	.	G	.	.	.	.	C	T
454	.	.	A	A	A	.	.	.	.	.	.	.	.	.	.	.	.	.	A	.	.	.	A	G	.	.	.	.	C	-
NL4-3	.	.	.	.	.	.	.	.	.	.	.	.	.	.	.	.	.	.	A	.	.	.	A	G	.	.	.	.	C	-

																									330				
89.6	-	-	-	-	-	-	-	-	-	-	-	-	-	-	-	-	-	-	-	-	A	C	T	G	C	T	G	A	C	A
409	-	-	-	-	-	-	-	-	-	-	-	-	-	-	-	-	-	-	-	-	-	-	-	-	-	.	.	.	.	.
421	G	A	C	A	T	C	G	A	-	-	-	-	G	A	A	C	A	C	A	A	.	.	.	.	.	.	.	.	.	.
439	G	A	C	A	C	T	G	A	G	C	T	C	C	T	A	C	A	A	G	G	.	.	.	.	.	.	.	.	.	.
454	-	-	-	-	-	-	-	-	-	-	-	-	-	-	-	-	-	-	-	-	-	-	-	-	-	.	.	.	.	.
NL4-3	-	-	-	-	-	-	-	-	-	-	-	-	-	-	-	-	-	-	-	-	-	-	-	-	-	.	.	.	.	.

						340										350										360	361		362	
Region																NFκB-II														
89.6	C	T	G	A	G	C	T	A	T	C	T	A	C	A	A	G	G	G	A	C	T	T	T	C	C	G	C	-	T	G
409	G	C	.	.	.	T	.	T	.	.	.	.	.	.	.	.	.	.	.	.	.	.	.	.	.	.	.	-	.	.
421	T	C	.	.	.	A	.	C	.	.	.	G	.	.	.	.	.	.	.	.	.	.	.	.	.	.	.	-	.	.
439	.	.	.	.	.	.	.	T	.	.	.	.	.	.	.	.	.	.	.	.	.	.	.	.	.	.	.	T	.	.
454	T	C	.	.	.	.	.	T	.	.	.	.	.	.	.	.	.	.	.	.	.	.	.	.	.	.	.	-	.	.
NL4-3	T	C	.	.	.	.	.	T	.	.	.	.	.	.	.	.	.	.	.	.	.	.	.	.	.	.	.	-	.	.

							370			373	374		375					380										390		
Region	NFκB-I												Sp1-III													Sp1-II				
89.6	G	G	G	A	C	T	T	T	C	C	A	-	G	G	G	A	G	G	T	G	T	G	G	C	C	T	G	G	G	C
409	.	.	.	.	.	.	.	.	.	.	G	-	.	.	.	.	.	.	C	.	.	.	A	.	A	.	.	.	.	.
421	.	.	.	.	.	.	.	.	.	.	.	-	.	.	.	.	.	.	C	.	.	.	A	.	.	.	.	.	.	.
439	.	.	.	.	.	.	.	.	.	.	.	-	.	.	.	.	.	.	A	.	.	.	.	.	.	.	.	.	.	.
454	.	.	.	.	.	.	.	.	.	.	.	G	.	.	.	.	.	.	.	.	.	.	.	.	.	.	.	.	.	.
NL4-3	.	.	.	.	.	.	.	.	.	.	.	-	.	.	.	.	.	.	.	.	.	.	.	.	.	.	.	.	.	.

				396												397			400										410	
Region	Sp1-II					Sp1-I																								
89.6	G	G	A	A	C	T	G	G	G	G	A	G	T	G	G	C	-	-	-	-	-	-	-	-	-	-	-	G	A	G
409	.	.	G	.	.	A	.	.	.	.	.	.	.	.	.	.	-	-	-	-	-	-	-	-	-	-	-	.	.	.
421	.	.	G	.	.	.	.	.	.	.	.	.	.	.	.	.	T	G	G	G	G	A	G	T	G	G	C	.	.	.
439	.	.	G	.	.	C	.	.	.	.	.	.	.	.	.	.	-	-	-	-	-	-	-	-	-	-	-	.	.	.
454	.	.	G	.	.	.	.	.	.	.	.	.	.	.	.	.	-	-	-	-	-	-	-	-	-	-	-	.	.	.
NL4-3	.	.	G	.	.	.	.	.	.	.	.	.	.	.	.	.	-	-	-	-	-	-	-	-	-	-	-	.	.	.

									420										430										440	
Region																TATA Box														
89.6	C	C	C	T	C	A	G	A	T	G	C	T	G	C	A	T	A	T	A	A	G	C	A	G	C	T	G	C	T	T
409	.	.	.	.	.	.	.	.	C	.	.	.	.	.	.	.	.	.	.	.	.	.	.	.	.	.	.	.	.	.
421	.	.	.	.	.	.	.	.	.	.	.	.	.	.	.	.	.	.	.	.	.	.	.	.	.	.	.	.	-	.
439	.	.	.	.	.	.	.	.	.	.	.	.	.	.	.	.	.	.	.	.	.	.	.	.	.	.	.	.	.	.
454	.	.	.	.	.	.	.	.	.	.	.	.	.	.	.	.	.	.	.	.	.	.	.	.	.	.	.	.	.	.
NL4-3	.	.	.	.	.	.	.	.	.	.	.	.	A	.	.	.	.	.	.	.	.	.	.	.	.	.	.	.	.	.

Supplementary Figure 5, continued

									450										460										470	
													TAR																	
89.6	T	T	T	G	C	C	T	G	T	A	C	T	G	G	G	T	C	T	C	T	C	T	G	G	T	T	A	G	A	C
409	.	.	.	.	.	.	.	.	.	.	.	.	.	.	.	.	.	.	.	.	.	.	A	.	.	.	.	.	.	.
421	.	.	.	.	.	.	.	.	.	.	.	.	.	.	.	.	.	.	.	.	.	.	.	.	.	.	.	.	.	.
439	.	.	.	.	.	.	.	.	.	.	.	.	.	.	.	.	.	.	.	.	.	.	.	.	.	.	.	.	.	.
454	.	.	.	.	.	.	.	.	.	.	.	.	.	.	.	.	.	.	.	.	.	.	.	.	.	.	.	.	.	.
NL4-3	.	.	.	.	.	.	.	.	.	.	.	.	.	.	.	.	.	.	.	.	.	.	.	.	.	.	.	.	.	.

									480										490										500	
	TAR																													
89.6	C	A	G	A	T	C	T	G	A	G	C	C	T	G	G	G	A	G	**C**	T	C	T	C	T	G	G	C	T	A	G
409	.	.	.	.	.	.	.	.	.	.	.	.	.	.	.	.	.	.	.	.	.	.	.	.	.	.	.	.	G	A
421	.	.	.	.	.	T	.	.	.	.	.	.	.	.	.	.	.	.	.	.	.	.	.	.	.	.	.	.	.	.
439	.	.	.	.	.	.	.	.	.	.	.	.	.	.	.	.	.	.	.	.	.	.	.	.	.	.	.	.	.	A
454	.	.	.	.	.	.	A	.	.	.	.	.	.	.	.	.	.	.	.	.	.	.	.	.	.	.	.	.	.	.
NL4-3	.	.	.	.	.	.	.	.	.	.	.	.	.	.	.	.	.	.	.	.	.	.	.	.	.	.	.	.	.	A

									510										520										530	
	TAR													NL4-3																
89.6	C	T	A	G	G	G	A	A	C	C	C	A	C	T	G	C	T	T	A	A	G	C	C	T	C	A	A	T	A	A
409	.	.	.	.	.	.	.	.	.	.	.	.	.	.	.	.	.	.	.	.	.	.	.	.	.	.	.	.	.	.
421	.	.	.	.	.	.	.	.	.	.	.	.	.	.	.	.	.	.	.	.	.	.	.	.	.	.	.	.	.	.
439	.	.	.	.	.	.	.	.	.	.	.	.	.	.	.	.	.	.	.	.	.	.	.	.	.	.	.	.	.	.
454	.	.	.	.	.	.	.	.	.	.	.	.	.	.	.	.	.	.	.	.	.	.	.	.	.	.	.	.	.	.
NL4-3	.	.	.	.	.	.	.	.	.	.	.	.	.	.	.	.	.	.	.	.	.	.	.	.	.	.	.	.	.	.

									540										550										560	
	NL4-3								AP1																					
89.6	A	G	C	T	T	G	C	C	T	T	G	A	G	T	G	C	T	T	C	A	A	G	T	A	G	T	G	T	G	T
409	.	.	.	.	.	.	.	.	.	.	.	.	.	.	.	.	.	C	A	.	.	.	.	.	.	.	.	.	.	.
421	.	.	.	.	.	.	.	.	.	.	.	.	.	.	.	.	.	C	A	.	.	.	.	.	.	.	.	.	.	.
439	.	.	.	.	.	.	.	.	.	.	.	.	.	.	.	.	.	C	A	.	.	.	.	.	.	.	.	.	.	.
454	.	.	.	.	.	.	.	.	.	.	.	.	.	.	.	.	.	C	A	.	.	.	.	.	.	.	.	.	.	.
NL4-3	.	.	.	.	.	.	.	.	.	.	.	.	.	.	.	.	.	C	A	.	.	.	.	.	.	.	.	.	.	.

									570										580										590	
	NL4-3									AP1																				
89.6	G	C	C	C	G	T	C	T	G	T	T	G	T	G	T	G	A	C	T	C	T	G	G	T	A	G	C	T	A	G
409	.	.	.	.	.	.	.	.	.	.	.	.	.	.	.	.	.	.	.	.	.	.	.	.	.	A	.	.	.	.
421	.	.	.	.	.	.	.	.	.	.	.	.	.	.	.	.	.	.	.	.	.	.	.	.	.	A	.	.	.	.
439	.	.	.	.	.	.	.	.	.	.	.	.	.	.	.	.	.	.	.	.	.	.	.	.	.	A	.	.	.	.
454	.	.	.	.	.	.	.	.	.	.	.	.	.	.	.	.	.	.	.	.	.	.	.	.	.	A	.	.	.	.
NL4-3	.	.	.	.	.	.	.	.	.	.	.	.	.	.	.	.	.	.	.	.	.	.	.	.	.	A	.	.	.	.

									600										610										620	
	NL4-3																													
89.6	A	G	A	T	C	C	C	T	C	A	G	A	C	C	A	T	C	T	T	A	G	T	T	A	G	T	G	T	A	G
409	.	.	.	.	.	.	.	.	.	.	.	.	.	.	.	.	T	.	.	.	.	.	C	.	.	.	.	.	G	.
421	.	.	.	.	.	.	.	.	.	.	.	.	.	.	T	C	T	.	.	.	.	.	C	.	.	.	.	.	G	.
439	.	.	.	.	.	.	.	.	.	.	.	.	.	.	C	.	T	.	.	.	.	.	C	.	.	.	.	.	G	.
454	.	.	.	.	.	.	.	.	.	.	.	.	.	.	C	.	T	.	.	.	.	.	C	.	.	.	.	.	G	.
NL4-3	.	.	.	.	.	.	.	.	.	.	.	.	.	.	C	.	T	.	.	.	.	.	C	.	.	.	.	.	G	.

										630										640										650
																PBS^{Lys3}														
89.6	-	A	A	A	A	T	C	T	C	T	A	G	C	A	G	T	G	G	C	G	C	C	C	G	A	A	C	A	G	G
409	-	.	.	.	.	.	.	.	.	.	.	.	T	.	.	.	.	.	.	.	.	.	.	.	.	.	.	.	.	.
421	-	.	.	.	.	.	.	.	.	.	.	.	.	.	.	.	.	.	.	.	.	.	.	.	.	.	.	.	.	.
439	-	.	.	.	.	.	.	.	.	.	.	.	.	.	.	.	.	.	.	.	.	.	.	.	.	.	.	.	.	.
454	A	.	.	.	.	.	.	.	.	.	.	.	.	.	.	.	.	.	.	.	.	.	.	.	.	.	.	.	.	.
NL4-3	-	.	.	.	.	.	.	.	.	.	.	.	.	.	.	.	.	.	.	.	.	.	.	.	.	.	.	.	.	.

Supplementary Figure 5, continued

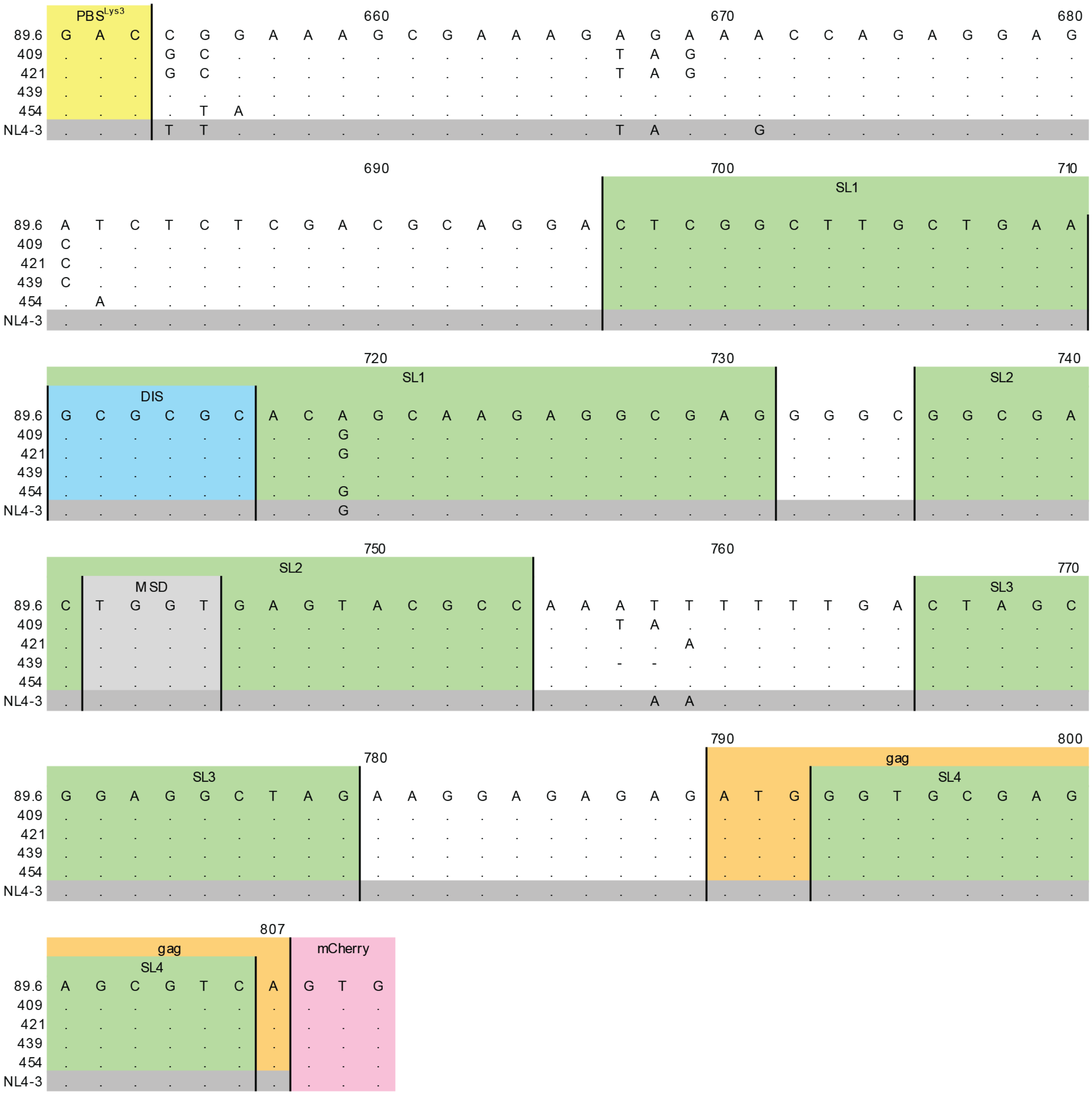

Supplementary Figure 5. LTR sequence variations in chimeric viruses. 5' LTR sequences derived from ART treated HIV patients and NL4-3 aligned relative to 89.6 VT1. Identical positions are marked with a dot, missing residues are denoted with "-". TAR C37 is in bold italic. Representative transcription factor binding sites, functional and regulatory regions and NL4-3 derived sequences are annotated with HXB2 position numbering from the Los Alamos National Laboratory HIV-1 2021 Compendium.

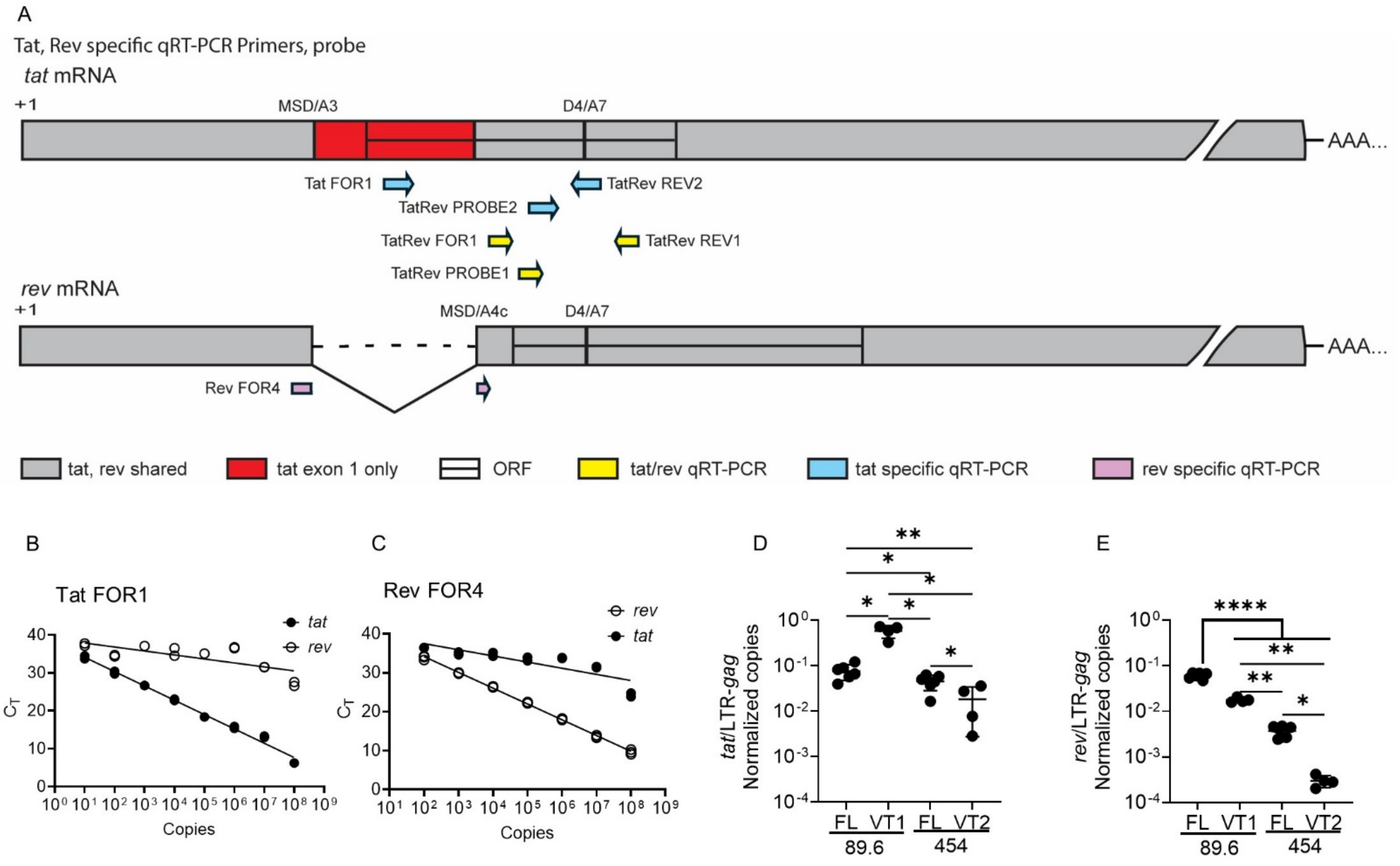

Supplementary Figure 6. Development of a qPCR assay that can distinguish *tat* and *rev* transcripts. (A) Schematic demonstrating the locations of RT-qPCR primers and probes. Relevant splice donor and acceptor sites are shown (MSD/A3 and D4/A7); MSD, major splice donor **(B, C)** Validation of primer-probe specificity for *tat* ***(B)*** and *rev* **(C)** selective RT-qPCR assays using the indicated synthetic template. Similar results were obtained in 3 experiments. **(D, E)** Summary graph of RT-qPCR analysis of RNA from transiently transfected HEK-293 T cells . Statistical significance was determined by One-way ANOVA with Tukey's multiple comparisons test. Mean values ± standard deviation are shown from 3-6 independent experiments, *P ≤ 0.05; **P ≤ 0.01; and ****P ≤ 0.0001. Reanalysis of the samples from Figure 5 F.

A Tat

a.a	1	2	3	4	5	6	7	8	9	10	11	12	13	14	15	16	17	18	19	20	21	22	23	24	25	26	27	28	29	30
89.6	M	E	P	V	N	P	S	L	E	P	W	K	H	P	G	S	Q	P	K	T	A	C	T	N	C	Y	C	K	K	C
454	-	-	-	-	D	-	R	-	-	-	-	-	-	-	-	-	-	-	R	-	-	-	N	-	-	-	-	-	-	-

a.a.	31	32	33	34	35	36	37	38	39	40	41	42	43	44	45	46	47	48	49	50	51	52	53	54	55	56	57	58	59	60
89.6	C	F	H	C	Q	A	C	F	I	T	K	G	L	G	I	S	Y	G	R	K	K	R	R	Q	R	R	R	P	P	Q
454	-	-	-	-	-	V	-	-	T	K	-	-	-	-	-	-	-	-	-	-	-	-	-	-	-	-	-	A	-	-

a.a.	61	62	63	64	65	66	67	68	69	70	71	72	73	74	75	76	77	78	79	80	81	82	83	84	85	86	87	88	89	90
89.6	D	S	Q	T	H	Q	V	S	L	S	K	Q	P	S	S	Q	P	R	G	D	P	T	G	P	K	E	Q	K	K	K
454	-	-	-	S	-	-	A	-	-	-	-	-	S	A	P	-	L	-	-	-	-	-	-	Q	-	-	S	-	-	E

a.a.	91	92	93	94	95	96	97	98	99	100	101
89.6	V	E	R	E	T	E	T	D	P	V	H
454	-	-	T	K	-	-	-	-	-	-	P

B

C

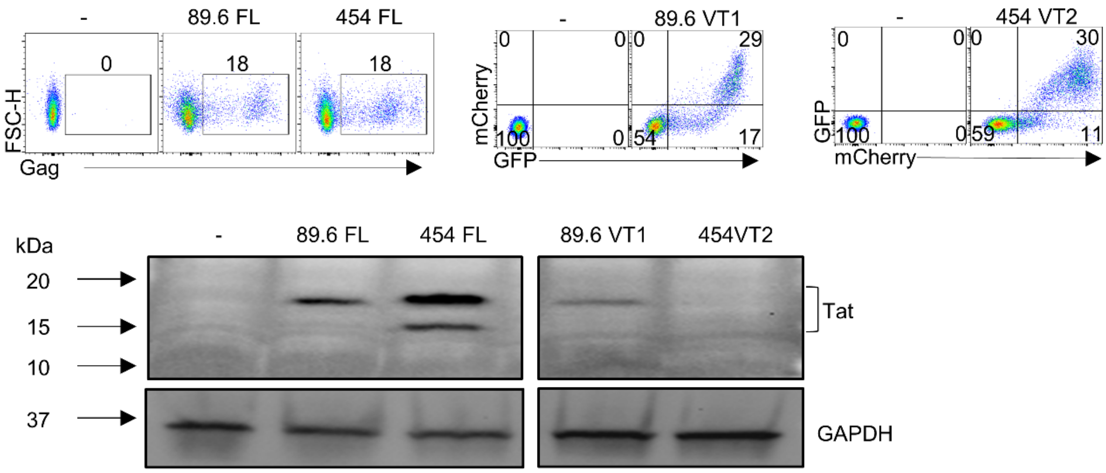

Supplementary Figure 7. Western blot analysis of HIV Tat expression. (A) Amino acid alignment of 89.6 and 454 Tat proteins. The epitope recognized by the antibody used in part (B) (PRLEPWKH) is boxed; 89.6 has a mismatch at the second position (P**S**LEPWKH). **(B)** Flow cytometric analysis of HEK-293T cells transiently transfected with the indicated virus or reporter construct as indicated. **(C)** Representative immunoblot analysis of HEK-293T cells from part B probed with an antibody directed against the indicated protein. Similar results were obtained in 4 experiments.

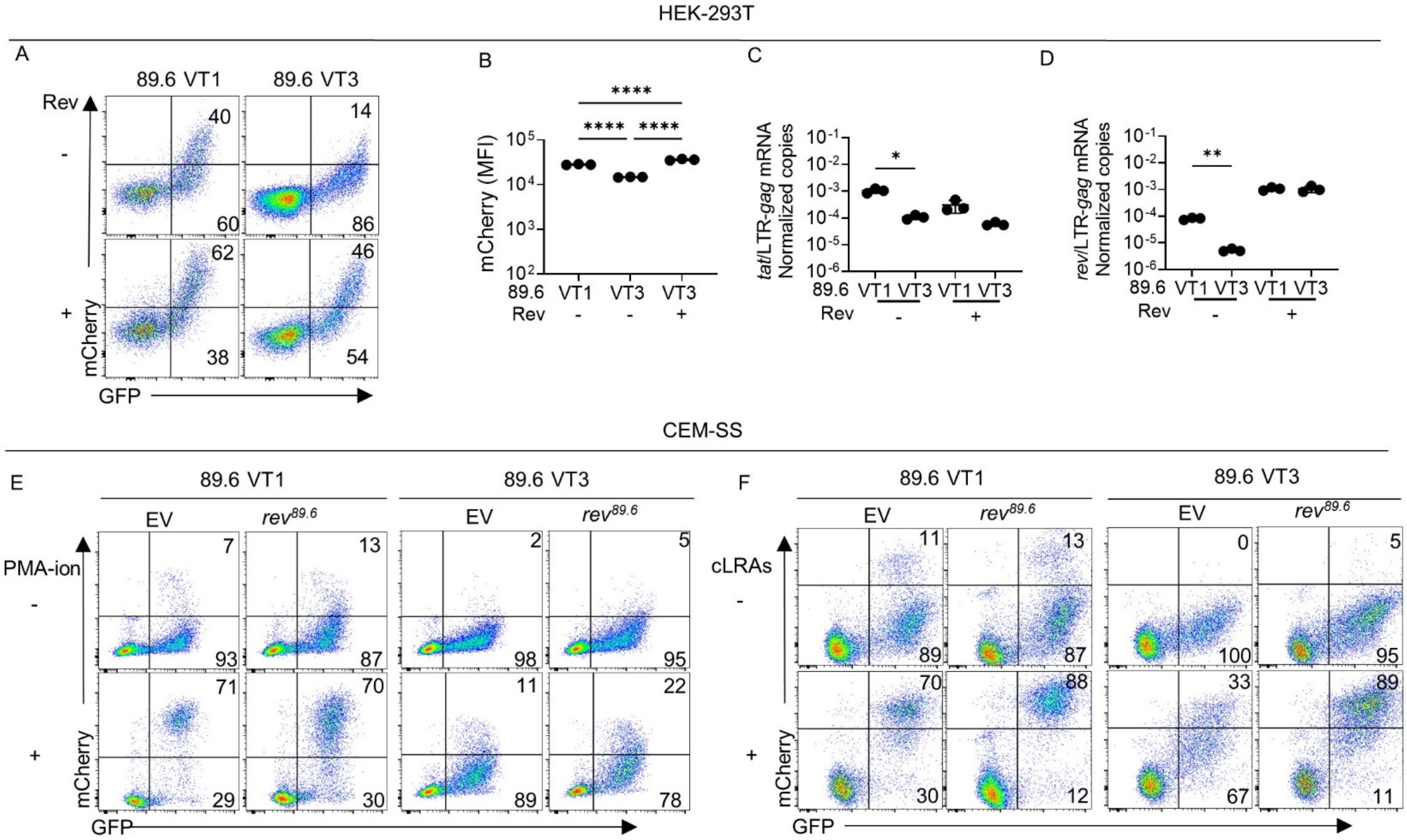

Supplementary Figure 8. Effect of exogenous Rev expression on 89.6 VT1 and 89.6 VT3 mCherry expression. **(A)** Flow cytometric analysis of HEK-293T cells transiently transfected with the indicated reporter construct plus a plasmid expressing *rev*$^{89.6}$ as indicated. **(B)** Summary graph of mCherry mean fluorescence intensity (MFI) for cells treated as in part (A). **(C-D)** RT-qPCR analysis of RNA isolated from HEK-293T cells transiently transfected as for part (A). **(B–D)** Statistical significance was determined by One-way ANOVA with Tukey's multiple comparisons test comparisons. Mean values ± standard deviation are shown from 3 independent experiments, *P ≤ 0.05; **P ≤ 0.01; ****P ≤ 0.0001 **(E-F)** Flow cytometric analysis of the indicated CEM-SS cell line stably expressing empty vector (EV) or *rev*$^{89.6}$, transduced with the indicated reporter virus, treated 2 dpi as indicated with (E) PMA-ionomycin or (F) cLRAs and harvested 3 dpi. Similar results were obtained in 3 independent experiments.

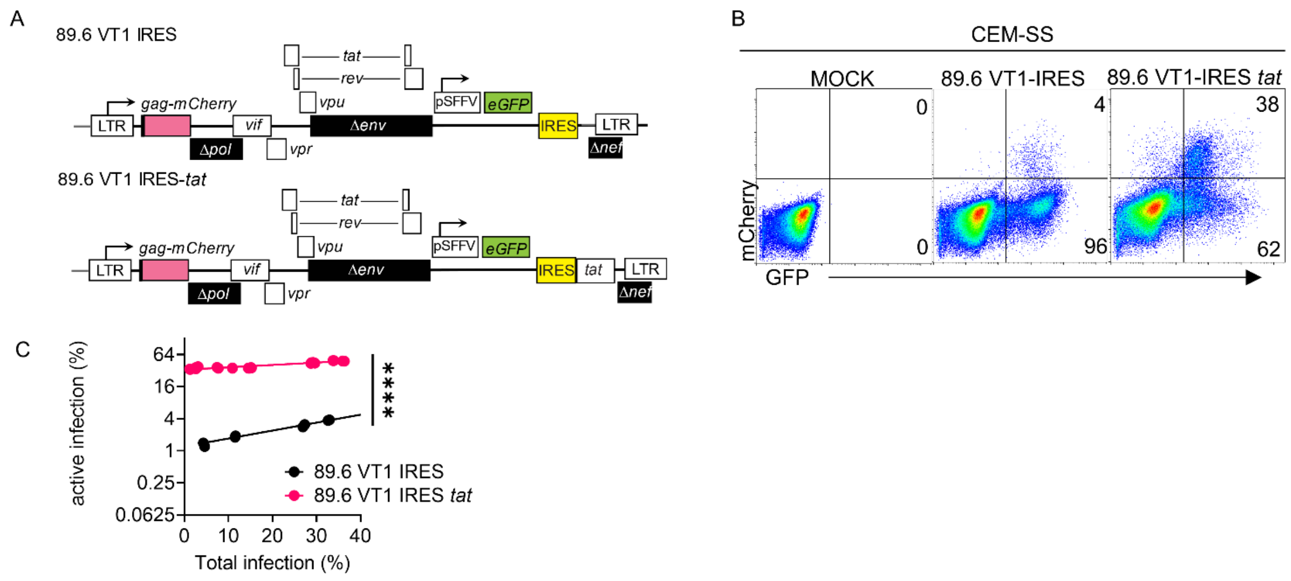

Supplementary Figure 9: Overexpression of HIV *tat* increases the proportion of active versus latent infection at the time of integration. (A) Diagram for dual reporters in which 89.6 VT1 (Figure 1A) was modified to include an internal ribosome entry site (IRES) plus or minus an HIV *tat* cassette 3' to the *eGFP* open reading frame. **(B)** Flow cytometric analysis of CEM-SS cells transduced with the indicated reporter virus and harvested 3 dpi. **(C)** Summary graph of experiment performed as in **(B)** with increasing amounts of the indicated virus and harvested 3 dpi. Each point represents a technical replicate from 3 independent experiments. Statistical significance was determined by Deming (Model II) linear regression, ****P ≤ 0.0001.

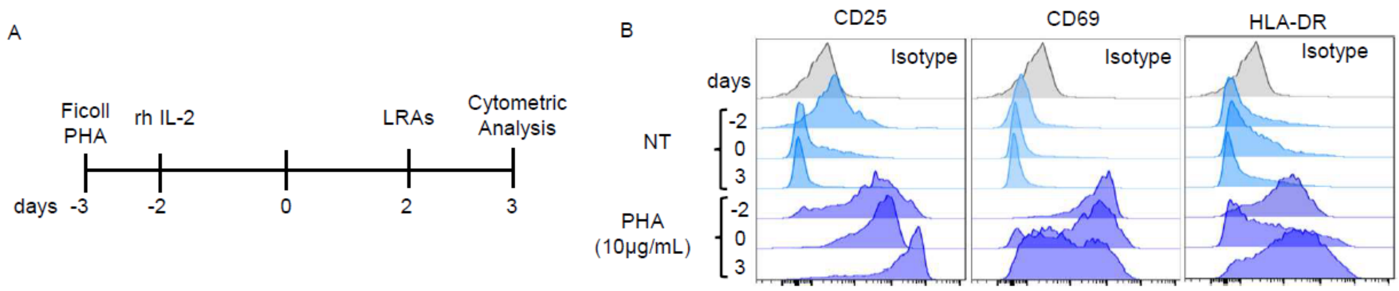

Supplementary Figure 10: Phenotypic expression of surface activation markers on primary CD4 T cells. (A) Schematic of the experimental process for (B). **(B)** Flow cytometric analysis of primary CD4$^+$ T cells treated as indicated with phytohemagglutinin (PHA) and stained with antibodies directed against the indicated activation marker according to the timeline shown in (A). PHA treated cells (3 days) were used for the isotype control. Similar results were obtained from 3 independent donors.

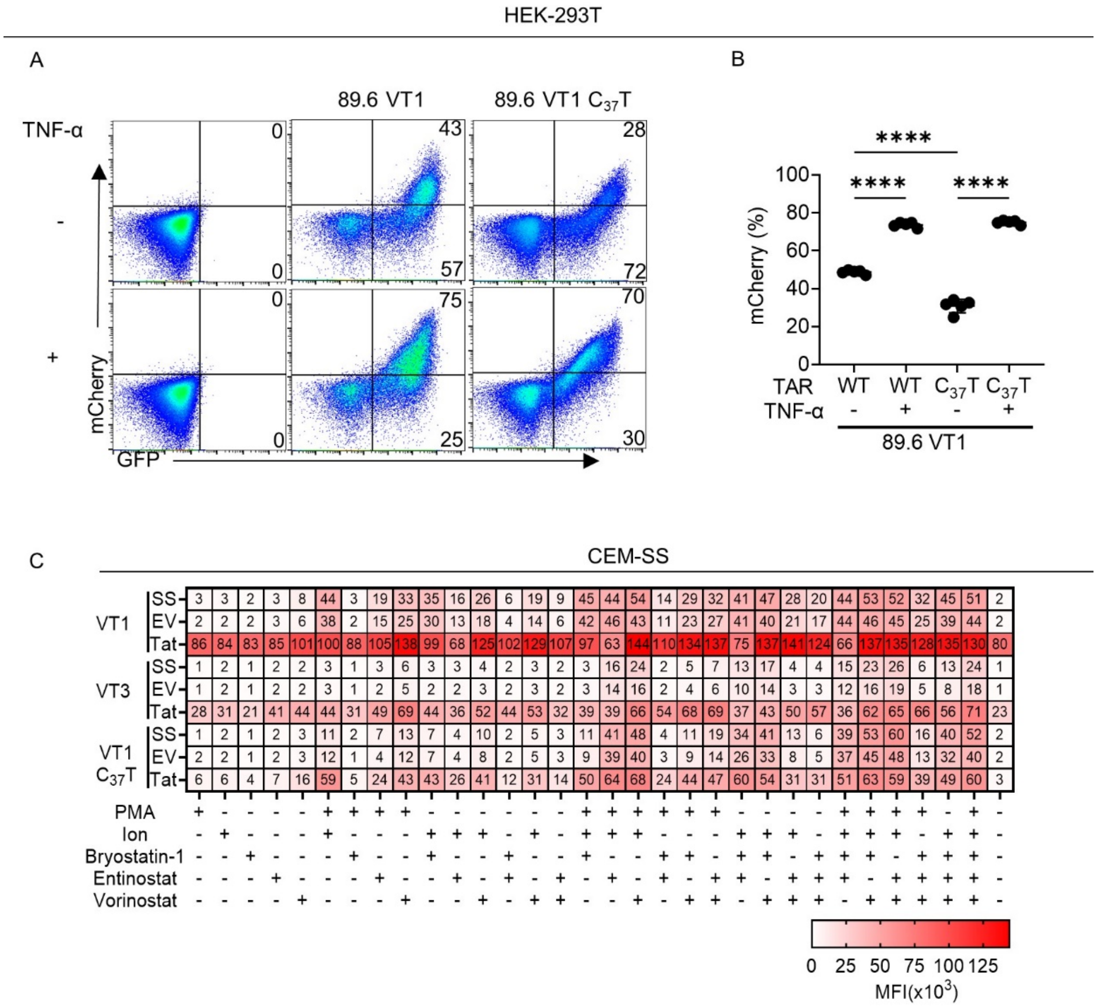

Supplementary Figure 11: Combinations of multiple latency reversal agents can ultimately reactivate latent HIV even when TAR is mutated. (A) Flow cytometric analysis of HEK-293T cells transiently transfected with the indicated construct and treated as indicated with TNF-α. **(B)** Summary graph of flow cytometric analysis of HEK-293T cells transiently transfected with the indicated plasmid and treated as indicated with TNF-α as in (A). Statistical significance was determined by One-way ANOVA with Šídák's multiple comparisons test. The mean ± SD represents 5 independent experiments, ****P $\leq$ 0.0001. **(C)** Heat map indicating the mean values for median fluorescence intensity (MFI) of infected (GFP^+) cells for the indicated cell type transduced with the indicated reporter virus and treated at 2 dpi with the indicated LRA. Cells were evaluated by flow cytometric analysis at 3 dpi. The mean MFI value from three independent experiments is shown (SS, parental CEM-SS cells; EV, CEM transduced with empty vector; Tat, CEM-**SS** transduced with a lentivirus expressing *tat*) .

Supplementary References:

1. Pear WS, Nolan GP, Scott ML, and Baltimore D. Production of high-titer helper-free retroviruses by transient transfection. *Proc Natl Acad Sci U S A.* 1993;90(18):8392-6.
2. Painter MM, Zaikos TD, and Collins KL. Quiescence Promotes Latent HIV Infection and Resistance to Reactivation from Latency with Histone Deacetylase Inhibitors. *Journal of Virology.* 2017;91(24).
3. Zaikos TD, Terry VH, Sebastian Kettinger NT, Lubow J, Painter MM, Virgilio MC, et al. Hematopoietic Stem and Progenitor Cells Are a Distinct HIV Reservoir that Contributes to Persistent Viremia in Suppressed Patients. *Cell Reports.* 2018;25(13):3759-73.e9.
4. Savell KE, Bach SV, Zipperly ME, Revanna JS, Goska NA, Tuscher JJ, et al. A Neuron-Optimized CRISPR/dCas9 Activation System for Robust and Specific Gene Regulation. *eneuro.* 2019;6(1):ENEURO.0495-18.
5. Yurkovetskiy L, Guney MH, Kim K, Goh SL, McCauley S, Dauphin A, et al. Primate immunodeficiency virus proteins Vpx and Vpr counteract transcriptional repression of proviruses by the HUSH complex. *Nature Microbiology.* 2018;3(12):1354-61.
6. Mashiba M, Collins David R, Terry Valeri H, and Collins Kathleen L. Vpr Overcomes Macrophage-Specific Restriction of HIV-1 Env Expression and Virion Production. *Cell Host & Microbe.* 2014;16(6):722-35.
7. Carter CC, Onafuwa-Nuga A, McNamara LA, Riddell J, Bixby D, Savona MR, et al. HIV-1 infects multipotent progenitor cells causing cell death and establishing latent cellular reservoirs. *Nature Medicine.* 2010;16(4):446-51.
8. O'Doherty U, Swiggard WJ, and Malim MH. Human immunodeficiency virus type 1 spinoculation enhances infection through virus binding. *J Virol.* 2000;74(21):10074-80.
9. Hilton HG, and Parham P. Direct binding to antigen-coated beads refines the specificity and cross-reactivity of four monoclonal antibodies that recognize polymorphic epitopes of HLA class I molecules. *Tissue Antigens.* 2013;81(4):212-20.
10. McNamara LA, Ganesh JA, and Collins KL. Latent HIV-1 infection occurs in multiple subsets of hematopoietic progenitor cells and is reversed by NF-κB activation. *J Virol.* 2012;86(17):9337-50.
