# Supplementary material for: Variation in HIV-1 Tat activity is a key determinant in the establishment of latent infection
Source: JCI Insight. 2024 Dec 5;10(2):e184711. doi: 10.1172/jci.insight.184711 (PMC11790021; doi:10.1172/jci.insight.184711)
Supplement: Unedited blot and gel images [file jciinsight-10-184711-s067.pdf]

Supplemental Figure 4A
Uncropped western blot

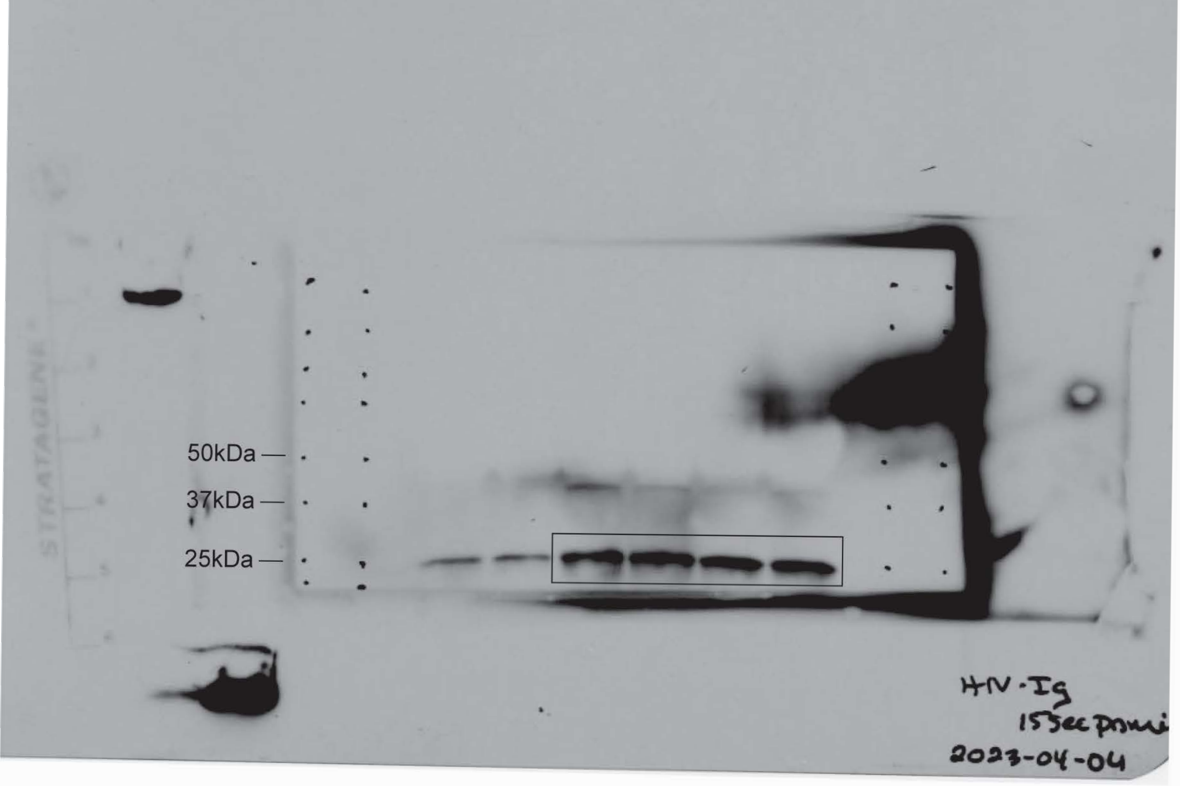

α-HIV-Ig
gag p24

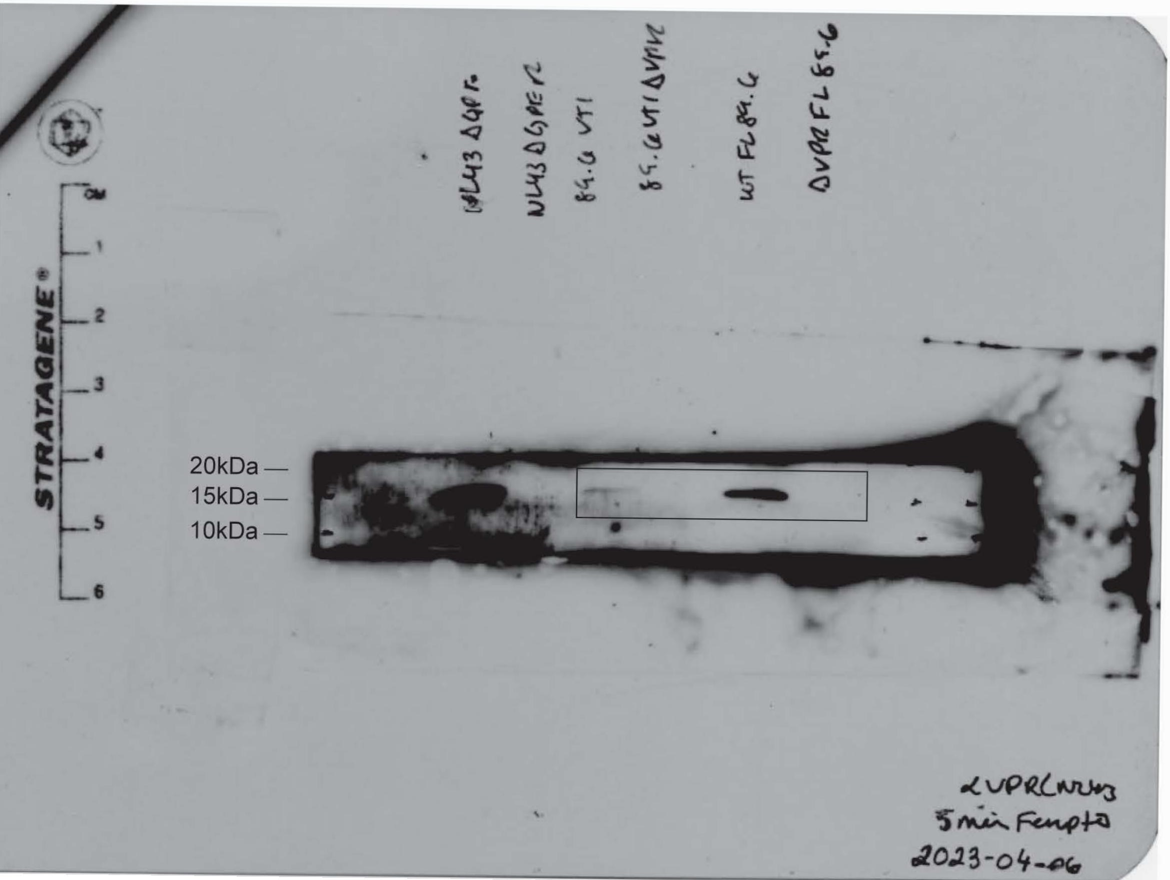

α-Vpr

Supplemental Figure 7C
Uncropped western blot

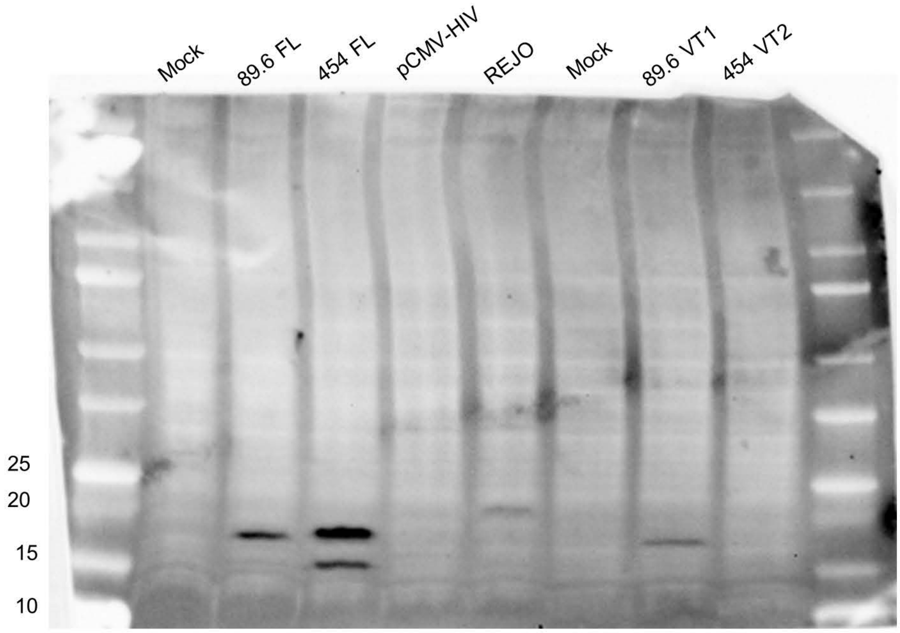

Thermo Tat Antibody 1:500

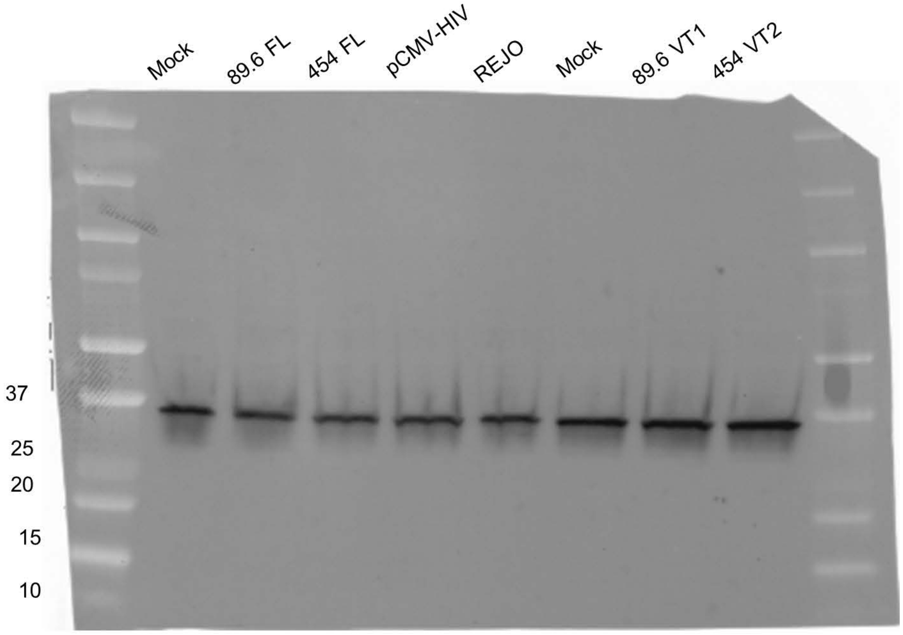

GAPDH
